# Sea-level rise exponentially increases coastal flood frequency

**DOI:** 10.1038/s41598-020-62188-4

**Published:** 2020-04-16

**Authors:** Mohsen Taherkhani, Sean Vitousek, Patrick L. Barnard, Neil Frazer, Tiffany R. Anderson, Charles H. Fletcher

**Affiliations:** 1 University of Illinois at Chicago, Department of Civil & Materials Engineering, Chicago, IL 60607 USA; 2 U.S. Geological Survey, Pacific Coastal and Marine Science Center, Santa Cruz, CA 95060 USA; 3 University of Hawai’i at Mānoa, School of Ocean and Earth Science and Technology, Department of Earth Sciences, Honolulu, HI 96822 USA

**Keywords:** Climate-change impacts, Natural hazards, Physical oceanography

## Abstract

Sea-level rise will radically redefine the coastline of the 21^st^ century. For many coastal regions, projections of global sea-level rise by the year 2100 (e.g., 0.5–2 meters) are comparable in magnitude to today’s extreme but short-lived increases in water level due to storms. Thus, the 21^st^ century will see significant changes to coastal flooding regimes (where present-day, extreme-but-rare events become common), which poses a major risk to the safety and sustainability of coastal communities worldwide. So far, estimates of future coastal flooding frequency focus on endpoint scenarios, such as the increase in flooding by 2050 or 2100. Here, we investigate the continuous shift in coastal flooding regimes by quantifying continuous rates of increase in the occurrence of extreme water-level events due to sea-level rise. We find that the odds of exceeding critical water-level thresholds increases exponentially with sea-level rise, meaning that fixed amounts of sea-level rise of only ~1–10 cm in areas with a narrow range of present-day extreme water levels can double the odds of flooding. Combining these growth rates with established sea-level rise projections, we find that the odds of extreme flooding double approximately every 5 years into the future. Further, we find that the present-day 50-year extreme water level (i.e., 2% annual chance of exceedance, based on historical records) will be exceeded annually before 2050 for most (i.e., 70%) of the coastal regions in the United States. Looking even farther into the future, the present-day 50-year extreme water level will be exceeded almost every day during peak tide (i.e., daily mean higher high water) before the end of the 21^st^ century for 90% of the U.S. coast. Our findings underscore the need for immediate planning and adaptation to mitigate the societal impacts of future flooding.

## Introduction

Sea-level rise is slow, yet consequential^1^ and accelerating^2^. Upper-end sea-level rise scenarios could displace hundreds of millions of people by the end of the 21^st^ century^3^. However, even small amounts of sea-level rise can disproportionately increase coastal flood frequency^4,5^. A multitude of oceanic processes affect both mean and extreme water levels, such as the tide, tropical and extratropical storms, climatic cycles (e.g., El Nino/Southern Oscillation), oceanic eddies, and circulation patterns^6–11^. Hence, the frequency and severity of coastal flooding varies on a multitude of time scales. Yet, the persistent trend and acceleration of sea-level rise have a profound interaction with transient extreme events^12^. In theory, sea-level rise progressively increases the frequency and severity of flooding^5^. In practice, the monotonic increase in flooding, driven by elevating long-term mean sea level, is often overshadowed by interannual variability in extreme events^13^, which will likely continue through the middle of the 21^st^ century^14^.

Many have quantified future increases in potential coastal flood frequency by deriving ‘multiplying factors’^15^, ‘amplification factors’^16^, or ‘factors of increase’^5^ in exceedance probability or, equivalently, reductions in return period of extreme water-level events due to sea-level rise by 2050 or 2100^13,17,18^. Large-scale studies of future ‘flooding’ typically investigate potential increases in the water-level hazard in the absence of site-specific exposure (as is the case in the current paper as discussed below in Application). The reported factors of increase in flood hazard potential are often exceedingly large, ranging from 10 to 1000 for even modest sea-level rise scenarios of 0.5 m or less. Yet, focusing on SLR scenarios and their impacts by 2050 or 2100 is perhaps inappropriate, given that significant changes in coastal flooding have been observed in recent years^19,20^ and are expected to change dramatically in the coming decades^5,21–23^, and planning horizons rarely exceed thirty years. While the incremental (e.g., ‘stair-cased’) factors of increase are staggering, they do not effectively illuminate the continuous, time-dependent transition between present and future flood hazard regimes that we will inevitably experience in the coming decades.

Taking a new approach, Sweet *et al*. (2017)^24^ and Stephens *et al*. (2018)^25^ identified the decade in the future that present-day, extreme water-level events become common-place (e.g., ~5 times per year). They found that many coastal cities transition to dramatically higher flood hazard regimes before 2050 for even moderate sea-level rise scenarios based on probabilistic projections. This new approach, establishing new flood hazard regimes based on a calendar date, effectively communicates the urgency for sea-level rise planning and adaptation. This concept has been further expanded by introducing the concept of a ‘trigger’^22^ or an ‘adaptation pathway’^26,27^, i.e., the combination of an intermediate sea-level threshold and an associated time frame when decisions must be made, in order to provide sufficient lead-time to efficiently adapt in a cost-effective manner before more critical flood hazard thresholds are exceeded.

Here, we estimate the timing of dramatic shifts in coastal flooding frequency by considering the rate of increase in flood frequency. We argue that the rate of flooding increase is a critical yet poorly understood component to address future sea-level rise impacts: sea-level rise is a continuous process, and thus increment-based assessments may misrepresent the underlying issue. As considered in previous works^15,28^ and based on the theoretical arguments presented in Methods, we focus on characterizing exponential rates of increase in extreme events driven by persistent shifts in mean water level due to sea-level rise. Note that an exponential increase implies a doubling in the frequency of extreme events over a given amount of sea-level rise or a given period of time, although the particular form of exponential growth used here has not been explicitly considered until now. Given well-established sea-level projections^29^ and water-level records at a number of long-running tide gauges around the U.S., we estimate sea-level and time scales associated with doubling the odds of exceeding extreme water-level thresholds, defined here as the present-day 50-year return level (i.e., 2% annual chance of exceedance; see Methods for details). For the most susceptible sites around the U.S., we find that the odds of exceeding extreme water-level thresholds are likely to double approximately every 5 years into the foreseeable future.

## Application

**Figure 1:**
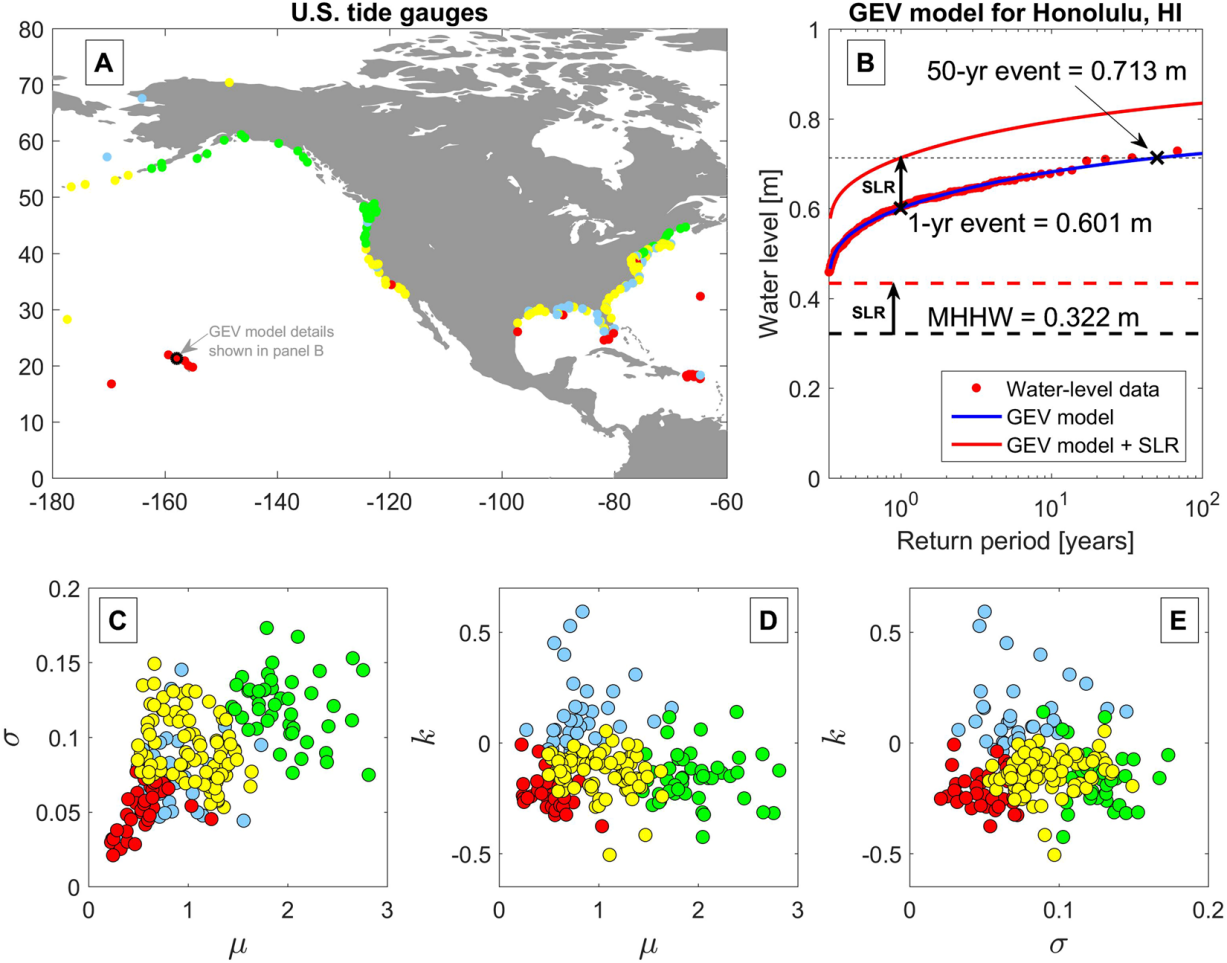
The network of 202 long-standing tide gauges used in the current study (**A**) and an example of the extreme water-level data and GEV fit for a single station at Honolulu, HI (**B**). (**C–E**) show ‘pair plots’ depicting the mutual relationship between the three best-fit parameters of the GEV distribution (*μ*, *σ*, and *k*) for each of the 202 tide stations used here. The color of each tide station depicts the K-means cluster to which each station belongs (i.e., red, yellow, green, or blue).

We utilize water-level observations at the long-standing network of tide gauges around the U.S. (obtained from the National Oceanic and Atmospheric Association (NOAA) Tides and Currents database, https://tidesandcurrents.noaa.gov/) to predict changes in future flood regimes due to sea-level rise. Although hundreds of tide stations exist around the U.S., we limit our analysis to 202 long-standing tide stations (Fig. 1A), based on the criteria described in Methods. In general, most tide gauges are located in harbors and embayments and thus are sheltered from the effects of waves. Hence, the analysis presented here is valid for the many sheltered coastal cities that are not directly exposed to wave setup, runup, and overtopping. In the current study, we focus our analysis on extreme water-level events (due to tides and storm surge but not waves) at the present-day 50-year return period, since most coastal engineering works in the U.S. are designed for return periods of 50 years or less^30^.

As is common among many large-scale assessments of sea-level rise impacts, we use extreme water level as a proxy representing coastal flood hazard potential, while acknowledging that site-specific flooding results from the complex interaction of extreme water levels, topography, and the built environment (e.g., coastal defense structures and drainage systems). For large-scale assessments of sea-level rise impacts, the practice of linking extreme water levels and coastal flooding is common throughout the scientific literature^5,13,15–18^, despite the fact that high-resolution topographic data and computationally onerous modeling efforts are required to properly characterize site-specific exposure to flood hazards^31^ (which are not considered here).

Using maximum likelihood estimates, we fit Generalized Extreme Value (GEV) probability distributions to the top three annual maxima of hourly water-level events at each tide gauge (See Methods; Fig. 1B). The GEV distribution models the probability of ‘block’ maxima, i.e., the maxima of a random variable occurring in a fixed time interval. The GEV distribution [see Eq. (7) in Methods] is defined in terms of three parameters *μ*, σ, and *k*, which represent the location (i.e., characteristic value), scale (i.e., characteristic width or variability), and shape (i.e., family type) of the distribution, respectively^32^. In the following analysis, we use the GEV model to categorize the extreme water-level behavior based on these three representative parameters. Later, we examine continuous rates of growth in extremes due to sea-level rise using empirical distributions of water levels rather than a particular statistical model like GEV.

Figure 1 shows the network of tide stations used in the current study (panel A) and an example of a GEV fit to the top 3 annual maxima water-level events observed at the Honolulu, HI tide gauge (panel B). Panel B plots the relationship between the magnitude of extreme water level and the expected return period, *T*_*R*_, which is given by Eq. (12) in Methods. Figure 1 panels C, D, and E show ‘pair plots’ depicting the mutual relationship between the three best-fit parameters of the GEV distribution (i.e., *μ*, σ, and *k*) for each station. Among the GEV parameters, we find only a notable relationship between the first two parameters, *μ* and σ (i.e., panel C), with a Pearson correlation coefficient of 0.55. For all the sites, we apply the K-means algorithm^33^ to cluster the GEV parameters into four groups (Fig. 1A,C–E) with distinct parameter values that characterize the behavior of extreme water level. Although the stations are not directly classified based on their geographic location, the clusters do exhibit consistent geographical patterns due to the underlying pattern of the water-level hazard.

In this study, the site-specific thresholds representing dramatic shifts in coastal flood regimes were determined by the difference in extreme water level, namely the Δ*Water Level *(Δ*WL*), between the 50-year and 1-year extreme water level, denoted by Δ*WL*_50yr→1yr_ (see e.g., Fig. 1B and Eq. (21) in Methods). We also determine Δ*WL*_50yr→MHHW_, which is the difference between the 50-year extreme water level and Mean Higher High Water (MHHW), i.e., the water level associated with the daily peak high tide (Fig. 1B; Eq. (22)). Although there are many analogous water-level metrics for the study of coastal flooding, we focus on the value of Δ*WL*_50yr→1yr_ because it represents a regime shift from a ‘once-in-a-lifetime’ occurrence (or rather, a ‘once-in-the-design-lifetime’ of an engineering structure) to an annual occurrence. Likewise, the Δ*WL*_50yr→MHHW_ metric for the 50-year water-level event becoming the MHHW event represents a regime shift from a ‘once-in-a-lifetime’ occurrence to a daily occurrence. The Δ*WL* metrics can be linked to sea-level rise; an amount of sea-level rise equal to Δ*WL*_50yr→1yr_ would cause the present-day 50-year water-level event to be exceeded every year. For example, Δ*WL*_50yr→1yr_ for Honolulu is only 11 cm (Fig. 1B), which represents the difference between the *T*_*R*_ = 50 year water level (0.713 m) and the *T*_*R*_ = 1 year water level (0.601 m). Thus, a sea-level increase on the order of 11 cm would be expected to cause large changes in the return period of coastal flooding for this site.

**Figure 2:**
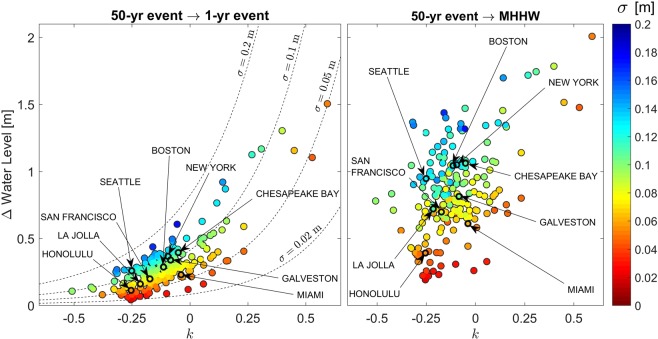
The Δ*Water Level* metrics for 202 long-standing tide stations in the U.S. (with labels provided for a few important tide stations). The Δ*WL*_50yr→1yr_ metric (left) and the Δ*WL*_50yr→MHHW_ metric (right) are shown as a function of the GEV shape parameter, *k*. The color of each tide station represents the magnitude of the GEV scale parameter, *σ*. In general, increasing *σ* and *k* increases Δ*WL*.

Figure 2 depicts Δ*WL*_50yr→1yr_ (left) and Δ*WL*_50yr→MHHW_ (right) as a function of the GEV shape parameter, *k*, for the 202 long-standing tide stations used in the current study (with labels provided for a few important tide stations). The color of each tide station shown in Fig. 2 represents the GEV scale parameter, *σ*, which often relates to the station’s susceptibility to sea-level rise, as discussed in the following section. The parameter *k* is a stronger factor in increasing Δ*WL*, particularly when *k* > 0 (as indicated by the contours of *σ* on Fig. 2). However, the majority of the sites (82%) have a negative shape parameter (i.e., *k* < 0). Δ*WL*_50yr→1yr_ exhibits a more obvious gradation with *σ* and *k* than Δ*WL*_50yr→MHHW_ (Fig. 2). This is likely because Δ*WL*_50yr→1yr_ is uniquely characterized by the GEV parameters, while Δ*WL*_50yr→MHHW_ is also influenced by the tidal characteristics, which are not fully described by the GEV parameters (since the GEV distribution only pertains to extreme water-level events). Nevertheless, there remains a clear relationship between the best-fit GEV parameters and both Δ*WL* metrics across each tide station: increasing *σ* and *k* increases Δ*WL*.

For more than 90% of our U.S. sites, the difference between the water level of the 50-year event and the 1-year event (i.e., Δ*WL*_50yr→1yr_) is less than 0.5 m. The difference in water level between the 50-year water-level event and MHHW (i.e., Δ*WL*_50yr→MHHW_) is on average approximately 0.85 m, with a standard deviation of 0.32 m. For 73% of the tide gauges used in this study, the difference in water level between the 50-year water-level event and the daily average highest tide (i.e., Δ*WL*_50yr→MHHW_) is less than 1.0 m. This is notable because most high-end sea-level rise projections exceed 1.0 m by 2100 (see e.g., Garner *et al*., (2018)^34^), which indicates that present-day extremes may occur daily in the future. Further, most low-end sea-level rise projections exceed 0.5 m by 2100^35^, indicating that present-day extremes may occur annually even in the best-case scenario.

**Figure 3:**
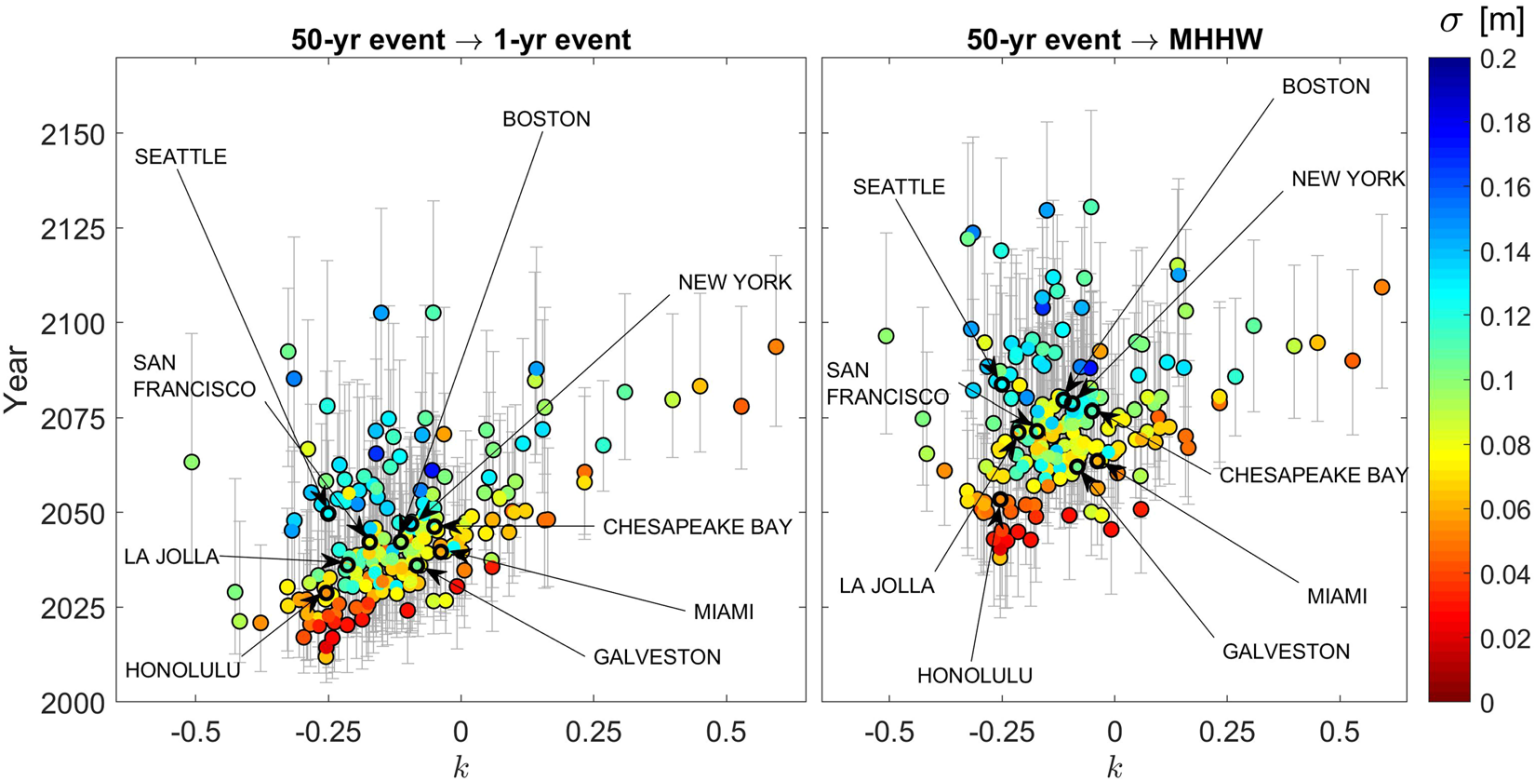
The year (y-axis) when the present-day 50-year water-event is exceeded annually (left), and, likewise, the year (y-axis) when the present-day 50-year water-level event is exceeded during a daily MHHW tide event (right), as a function of the GEV shape parameter, *k*, (on the x-axis) for the 202 tide stations used in the current study (with labels provided for a few important tide stations). As in Fig. 2, the color of each tide station represents the magnitude of the GEV scale parameter, *σ*. The results indicate when the sea-level rise projections, based on the RCP 8.5 emissions scenarios in Kopp *et al*., (2014)^29^, exceed the Δ*Water Level* results shown in Fig. 2. Each data point includes 95% confidence levels, which bound the probabilistic sea-level rise projections^29^. In general, increasing *σ* and *k* delays when the 50-year to 1-year and the 50-year to MHHW regime shifts occur.

Next, we apply spatiotemporally variable sea-level rise projections from Kopp *et al*. (2014)^29^ at each station to find the year in the future when sea-level rise exceeds the Δ*WL*_50yr→1yr_ and Δ*WL*_50yr→MHHW_ scenarios (Fig. 3). The sea-level rise projections are based on the Representative Concentration Pathways (RCP^36^) 8.5 (“business-as-usual”) emission scenario (two lower emissions scenarios, RCP2.6 and 4.5, are discussed later). Each data point includes 95% confidence levels representing the spread of the probabilistic sea-level rise projections from Kopp *et al*., (2014)^29^. In general, increasing *σ* and *k* delays the shifts of both flooding regime changes.

Focusing on the ensemble median sea-level rise scenarios, all of the labeled tide stations transition from 50-year water-level events to annual events before 2050. For nearly 70% of the tide stations used in this study, sea-level rise causes flood regime shifts from the present-day 50-year water-level event to an annual occurrence before the year 2050. 99% of tide stations in the U.S. transition from a 50-year occurrence to an annual occurrence before 2100. Considering the even more dire scenario of a flood regime transition from a 50-year occurrence to a near-daily occurrence (i.e., during a MHHW event), we find that only 6% of stations transition before 2050, but the number climbs to 62% before 2075. 93% of tide stations in the U.S. will transition from a 50-year recurrence of an extreme flooding event to a daily occurrence before 2100. The impact of this finding bears repeating: sea-level rise will likely cause ‘once-in-a-lifetime’ coastal flooding events to occur nearly every day before 2100.

Thus far, our analyses provide insights into the difference in water level between critical thresholds and the timing of apparent regime shifts. However, they do not illuminate the continuous rates of change in sea-level rise impacts, which are critical to understand since the effects foreshadowing these two regime shifts (which represent fairly dire scenarios) will be experienced much earlier.

**Figure 4:**
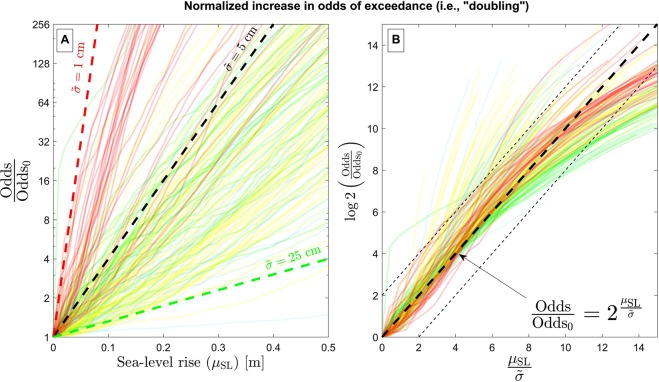
The relationship between sea-level rise and the relative increase in the odds of exceeding the present-day 50-year water-level event, *O*/*O*_0_. Each colored solid line corresponds to the relationship (*O*/*O*_0_ vs. sea-level rise) for a single tide station, and each station is colored according to its classification shown in Fig. 1 (however, some transparency is applied to each line to reduce occlusion). Panel A shows a plot of the relative odds *O*/*O*_0_ vs. the amount of sea-level rise (${\mu }_{{\rm{SL}}}$), where the x- and y-axes of the panel are on linear and (base-two) logarithmic scales, respectively. Hence, relationships that follow a straight line correspond to exponential growth (i.e., doubling) with a fixed amount of sea-level rise on the x-axis. The red, black, and green dashed lines on panel B correspond to doubling the of odds of exceedance with every 1 cm, 5 cm, and 25 cm of sea-level rise, respectively, according to Eq. (5) in Methods. Panel B shows a plot of the base-two logarithm of the relative odds, i.e., ${\log }_{2}(O/{O}_{0})$ vs. the normalized amount of sea-level rise (${\mu }_{{\rm{SL}}}/\tilde{\sigma }$), where the parameter $\tilde{\sigma }$ is derived from the average slope of the curves in panel A. The straight, one-to-one line (i.e., the thick dashed line) on panel B corresponds to the relationship $O/{O}_{0}={2}^{{\mu }_{{\rm{S}}{\rm{L}}}/\mathop{\sigma }\limits^{ \sim }}$ (from Eq. (5) in Methods). The thin dashed lines on Panel B enclose areas that are within a factor of 4 of the relationship in Eq. (5).

**Figure 5:**
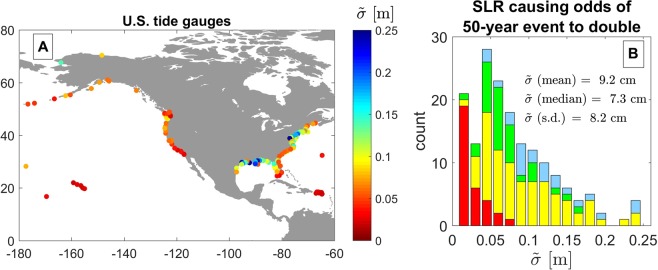
The variability of $\tilde{\sigma }$, the amount of sea-level rise that doubles the odds of exceeding the present-day 50-year water-level event according to Eq. (5) in Methods. Panel A shows the spatial variability of $\tilde{\sigma }$ at the tide stations used in the present study, and Panel B shows the histogram of $\tilde{\sigma }$ for all stations. The different colors (i.e., red, yellow, green, or blue) of the histogram in panel B depict the K-means cluster associated with the distinct groups of tide station as shown in Fig. 1.

Figures 4 and 5 analyze the rate of change in flood frequency due to sea-level rise. Figure 4 illustrates the relationship between sea-level rise and the relative increase in the odds of exceeding the present-day 50-year water-level event, *O*/*O*_*0*_. In Extended Data Figs. 1 and 2, we also explore the relationship between sea-level rise and the relative increase in the probability of exceeding the present-day 50-year water-level event, $E/{E}_{0}$. Here, we focus on the (rarely-used) metric of the increase in the odds of exceedance, *O*/*O*_0_, as opposed to the increase in exceedance probability, $E/{E}_{0}$, (considered previously^5,16^) as the odds increase is well suited to describe persistent rates of growth. Unlike exceedance probability (which is bounded with a probability of 1), the odds of flooding increases without bounds due to sea-level rise (see Extended Data).

We calculate the increased odds, $O=E/(1-E)$, from the future exceedance probability *E*, which is calculated from the present-day empirical exceedance probability distribution, ${E}_{0}(x)$, where *x* represents all values of recorded hourly water level for each individual tide station. Here, the future exceedance probability distribution is calculated by shifting the present-day distribution by a variable amount of sea-level rise, according to $E={E}_{0}(x-{\mu }_{{\rm{SL}}})$. This shift of the distribution corresponds to increasing the mean value of *x* by an amount ${\mu }_{{\rm{SL}}}$. In this case, the role of ${\mu }_{{\rm{SL}}}$ is equivalent to the role of sea-level rise, which increases the mean of the water-level distribution at a given site (see e.g., Fig. 8 in Methods). In the following analysis, we quantify growth rates in the odds of exceeding the 50-year extreme water-level threshold, however, similar growth rates can be quantified for arbitrary thresholds. Here, the 50-year extreme water-level threshold is calculated from the GEV distribution (see Methods, Eq. (12)) and the same threshold is applied to represent the 50-year extreme water level in the empirical distribution for the purposes of the following growth rate analysis.

**Figure 6:**
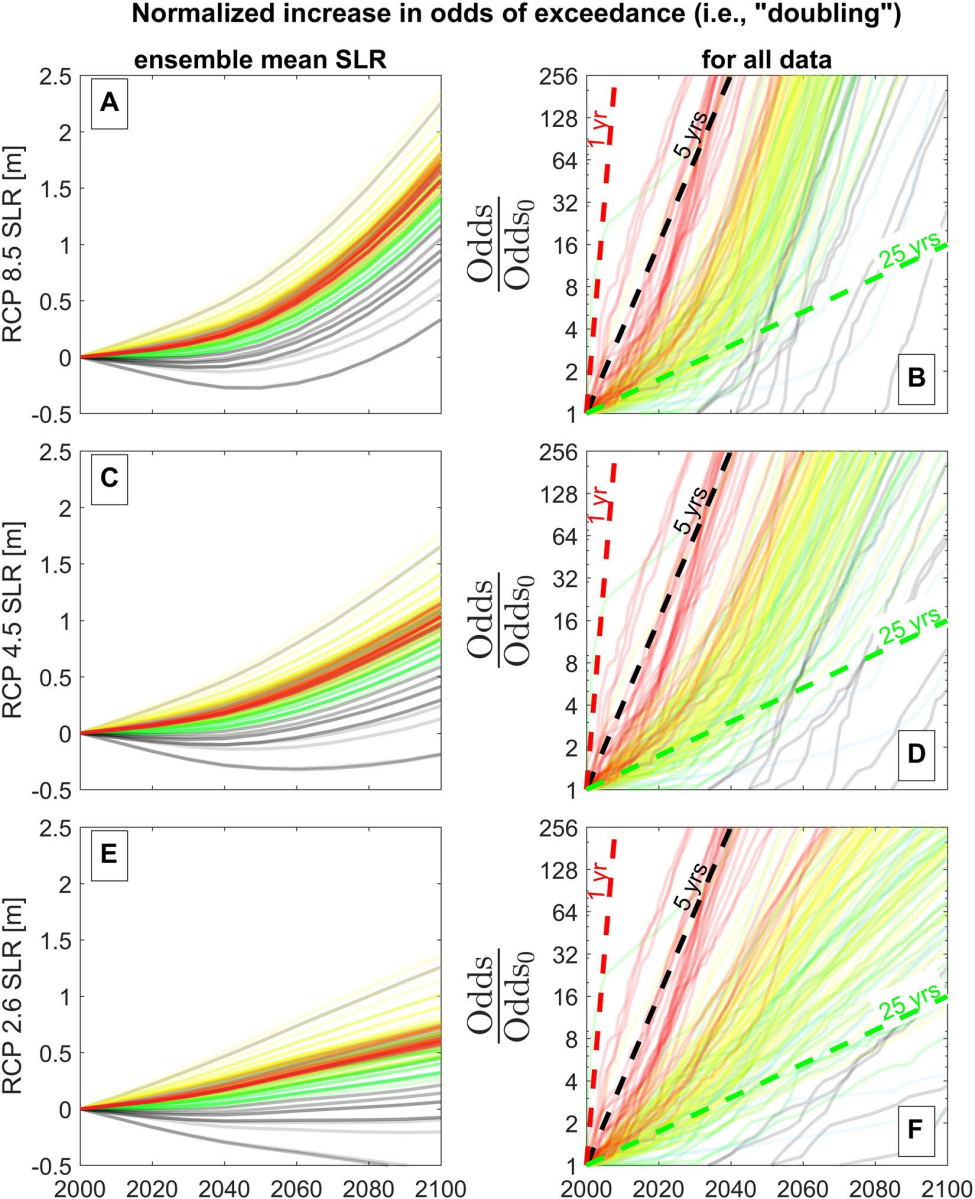
The relative increase in the odds of exceeding the present-day 50-year water-level event, *O*/*O*_0_, with respect to time. In this case, the odds increase is driven entirely by the sea-level rise scenarios of Kopp *et al*. (2014)^29^, which are shown in panels A, C, and E that correspond to RCP 8.5, 4.5, and 2.6 emissions scenarios, respectively. As in panel A of Fig. 4, panels B, D, and F calculate the odds from the empirical exceedance distribution resulting from all values of the recorded hourly water level. The x- and y-axes of panels B, D, and F are on linear and (base-two) logarithmic scales, respectively. Hence, relationships that follow a straight line correspond to exponential growth (i.e., doubling) with a fixed amount of time on the x-axis. Each solid line corresponds to the relationship between *O*/*O*_0_ and sea-level rise for a single tide station, and each station is colored according to its classification shown in Fig. 1 (however, some transparency is applied to each line to reduce occlusion). The black solid lines correspond to tide stations where sea-level rise is projected to decrease with time in the next few decades (which are generally located at high latitudes due to Glacio-isostatic adjustment). The red, black, and green dashed lines in panels B, D, and F correspond to a doubling of exceedance odds with every 1 yr, 5 yr, and 25 yr period, respectively, according to Eq. (6) in Methods.

Figure 4 panel A shows the results of the normalized increase in the odds of exceedance (y-axis) vs. sea-level rise (i.e., ${\mu }_{{\rm{SL}}}$ on the x-axis). The x- and y-axes of Fig. 4A are on linear and (base-two) logarithmic scales, respectively. Hence, relationships that follow straight lines correspond to exponential growth with sea-level rise on the x-axis. Each solid line on Fig. 4A corresponds to the relationship between *O*/*O*_0_ and sea-level rise for one of the 202 tide station used, which is colored according to its K-means cluster shown in Fig. 1. The red, black, and green dashed lines on Fig. 4A correspond to a doubling of exceedance odds with every 1 cm, 5 cm, and 25 cm of sea-level rise, respectively, according to Eq. (5). Hence, for susceptible, low-latitude sites (red clusters in Fig. 4), one centimeter of sea-level rise can cause a doubling of the exceedance odds at the 50-year water level threshold. Figure 4B shows a plot of the base-two logarithm of the relative odds, i.e., ${\log }_{2}(O/{O}_{0})$ vs. the normalized amount of sea-level rise (${\mu }_{{\rm{SL}}}/\tilde{\sigma }$), where the doubling parameter $\tilde{\sigma }$ is derived from the average slope of the curves in Fig. 4A. The straight, one-to-one line (i.e., the thick dashed line) on panel B corresponds to the relationship $\frac{O}{{O}_{0}}={2}^{\frac{{\mu }_{{\rm{SL}}}}{\tilde{\sigma }}}$ after Eq. (5) in Methods. The behavior of the exponential increase in odds of exceedance (evident in Fig. 4) is further discussed in the following section.

Here, we focus on results derived from the empirical exceedance distribution, ${E}_{0}(x)$, since it is nonparametric and, thus, the rates of increase do not depend on the behavior of any particular statistical model, e.g., the GEV model. Further, we apply an empirical distribution of all values of recorded water level, rather than a distribution representing the extreme water level, which is necessary when investigating the regime shifts from rare to commonplace events^37^. We compare three different representations of the exceedance distribution, ${E}_{0}(x)$: (1) the empirical distribution of all values of *x*, (2) the empirical distribution of extreme values of *x*, and (3) the best-fit GEV model for extreme values of *x* (see Extended Data Figs. 1 and 2). This comparison showed that, when calculating the odds increase, the rates of growth are fairly insensitive to the form of the exceedance distribution used.

Figure 5 shows the variability of $\tilde{\sigma }$, the amount of sea-level rise that doubles the odds of exceeding the present-day 50-year water-level event. Here, we derive $\tilde{\sigma }$ from the average slope of the trend between sea-level rise and the relative increase in the odds of exceeding the present-day 50-year water-level event (see Fig. 4A). Across all sites, $\tilde{\sigma }$ ranges between 1.9 cm and 27.5 cm with a mean of approximately 9.2 cm and a standard deviation of 8.2 cm (Fig. 5B).

Figure 6 illustrates the relative increase in the odds of exceeding the present-day 50-year water-level event, *O*/*O*_0_, with respect to time for different sea-level rise scenarios. The increases in odds are calculated from the ensemble median sea-level rise projections of Kopp *et al*., (2014)^29^ (depicted in the first column of Fig. 6, i.e., panels A, C, and E) at each tide station. The sea-level projections at each tide station are colored according to their classification shown in Fig. 1. The black solid lines on Fig. 6 correspond to a few tide stations where sea-level rise is projected to decrease with time in the short-term over the next few decades (which are mostly located at high latitudes) due to local land uplift. As in Fig. 4, the second column of Fig. 6 (e.g., panels B, D, and F) applies the empirical exceedance distribution resulting from all values of the recorded hourly water level to calculate the odds increase. We compare different representations of the exceedance distribution, ${E}_{0}(x)$, in Extended Data Figs. 3 and 4. Yet, we find that the rates of growth are somewhat insensitive to the form of the exceedance distribution used (see Extended Data Figs. 3 and 4). In general, the sites follow exponential behavior (i.e., doubling in a fixed amount of time) with subtle differences in growth rates associated with each sea-level projection, as discussed in the following section.

**Figure 7:**
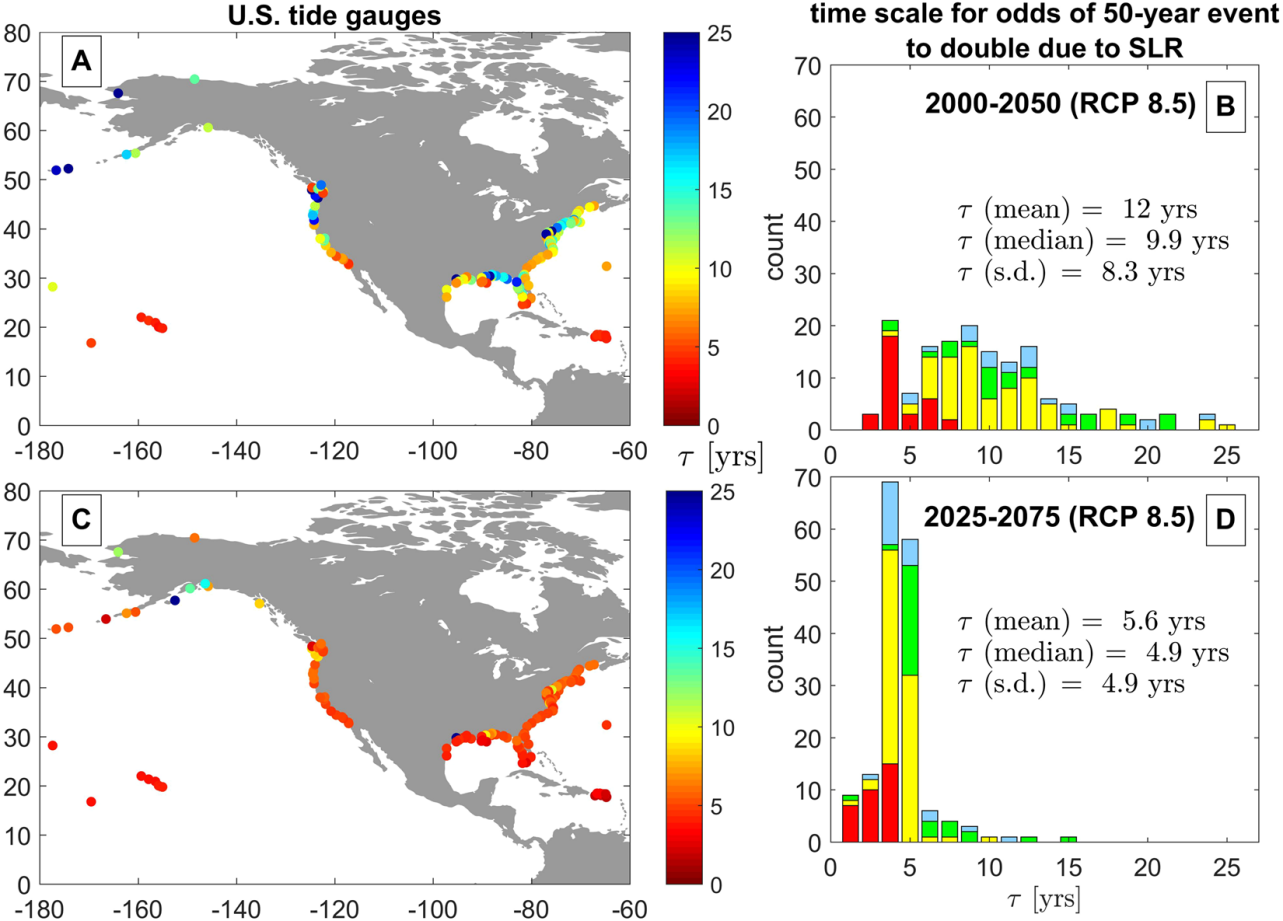
The spatial and temporal variability of the time scale, $\tau$, over which sea-level rise doubles the odds of exceeding the present-day 50-year water-level event, following Eq. (6) in Methods. Panels A and C show the spatial variability of $\tau$ at tide stations along the U.S. coast under the RCP 8.5 scenario for the time periods 2000–2050 and 2025–2075, respectively. Panels B and D show histogram plots of $\tau$ for the time periods 2000–2050 and 2025–2075, respectively.

Next, we examine the spatial and temporal variability of the time scale $\tau$, which represents the number of years until sea-level rise doubles the odds of exceeding the present-day 50-year water-level event (see Fig. 7 and Eq. 6 in Methods). Using the temporal trends in the odds of exceeding the present-day 50-year water-level event, *O*/*O*_0_ (Fig. 6B), $\tau$ is derived from the average slope of these curves. Panel A and C show the spatial variability of $\tau$ for the time period 2000–2050 and 2025–2075, respectively, under the RCP 8.5 scenario at the long-standing tide stations along the U.S. coast used in the current study. We selected the two time periods (2000–2050 and 2025–2075) to represent a present-day/near-future period and a future period centered on 2050, respectively, to demonstrate the role of accelerated sea-level rise on the doubling time. Across all sites, the median value of $\tau$ is approximately 9.9 years for the time period 2000–2050 (Fig. 7A,B), and decreases to 4.9 years for the time period 2025–2075 due to the acceleration of sea-level rise (Fig. 7C,D). The different colors (i.e., red, yellow, green, or blue) on Fig. 7B,D  depict the K-means cluster associated with the distinct groups of tide station as shown in Fig. 1. The spatial and temporal patterns of $\tau$ are discussed in the following section.

**Figure 8:**
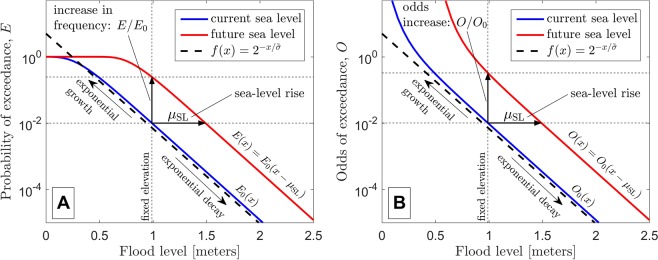
Schematic showing the current methodology to calculate the rate of growth in the exceedance probability and odds due to sea-level rise in panels A and B, respectively. Panels A and B both apply the GEV model with ${\mu }=0.3\,{\rm{m}},{\sigma }=0.15\,{\rm{m}},{k}=0.0,{{\mu }}_{{SL}}=0.5\,{\rm{m}}$ as motivating examples for the exponential rates of growth described in Eqs. (3) and (5), respectively. The growth rate parameter is given by $\tilde{{\sigma }}=0.7{\sigma }=0.105$ m in this example. As seen in panel A, the growth rate (${E}/{{E}}_{0}$) becomes bounded for large values of sea-level rise. On the other hand, the growth rate in the odds of exceedance (${O}/{{O}}_{0}$) is not bounded for large values of sea-level rise, as shown in panel B. Y-axes are on a logarithmic scale.

## Discussion

The parameters defining the GEV distribution at each station are location-dependent and spatially coherent, as indicated by the position of the K-means clusters shown in Fig. 1A. Similar to the geographic distribution of the GEV parameters shown in Vitousek *et al*. (2017)^5^ and Rueda *et al*. (2017)^38^, latitude exerts a strong influence on the cluster location, and more specifically, the value of the GEV scale parameter, $\sigma$. In general, the tropics experience small values of $\sigma$ (i.e., the red clusters in Fig. 1A), mid-latitudes experience intermediate values of $\sigma$ (i.e., the yellow clusters in Fig. 1A), and high-latitudes experience large values of $\sigma$ (i.e., the green clusters in Fig. 1A). The increase in $\sigma$ with latitude is largely driven by higher extratropical storm activity and tide ranges moving north along the U.S. coastline. It is well known that decreasing $\sigma$ increases the susceptibility to higher event frequencies with sea-level rise^5,15,38–40^. Hence, the red, yellow, and green clusters also generally represent high, medium, and low susceptibility to increases in flood frequency associated with sea-level rise, respectively. The gradient in $\sigma$ (and therefore the gradient in susceptibility to sea-level rise) with latitude is particularly evident on the U.S. West Coast (as shown in Fig. 1A). Conversely, the clustering of the GEV parameters for stations on the U.S. East Coast is less coherent, as the East Coast is punctuated by stations experiencing high values of the GEV shape parameter, $k$, (i.e., the blue clusters on Fig. 1A). As reported previously^5,38,41,42^, values of $k > 0$ are typically found in tropical cyclone regions, where exceedingly large yet rare wave heights and/or water-level events can occur. A minority (18%) of our sites had positive values of the GEV shape parameter, $k$, which corresponds to unbounded probability distributions, i.e., distributions with non-zero probabilities for events of arbitrarily large magnitude. This unbounded nature of extreme events is, of course, not physically realistic, but arises from the occurrence of outliers among data sets of limited duration.

Here, we consider a stationary approach to extreme value theory, meaning that the exceedance probability distributions (or parameters thereof) remain constant with time, except for the shifting mean due to sea-level rise. Non-stationary methods^43,44^, on the other hand, allow for extreme distributions (or their parameters) to vary with time, but typically require longer records of data. Devlin *et al*. (2017a,b, 2019)^45–47^ and Haigh *et al*. (2019)^48^ investigated non-stationary, non-astronomical changes in tidal amplitudes due to changes in mean sea level, which can alter the probabilities of exceeding flood levels during high‐tide events. Vousdoukas *et al*. (2018)^18^ investigated non-stationary changes in extreme water-level events at the 100-year return period due to climate model projections of different emissions scenarios. They found relatively small changes (<10 cm changes in absolute value) in the 100-year extreme water levels for most of the globe, except at high-latitudes. Furthermore, Vousdoukas *et al*., (2018)^18^ found that the contribution of non-stationary changes in extreme water level decreases rapidly relative to sea-level rise as a function of time and higher emission scenarios. Hence, we consider the effect of non-stationary changes to the mean of the extreme water-level distributions, but do not consider any non-stationary (i.e., time-dependent) changes to the distribution’s variance or skewness resulting from increases/decreases in storminess due to climate change or tide/sea-level interactions.

There is a strong geographic link between lower values of the scale, $\sigma$, and shape parameters, $k$, of the GEV distribution (red and yellow colors on Fig. 1) and increased susceptibility to sea-level rise impacts quantified via the $\Delta \,Water\,Level$ metrics (Fig. 2). The relationship between the sites’ shape parameter, $k$, and the amount of sea-level rise needed to shift flooding regimes (e.g., $\Delta \,Water\,Level$ in Fig. 2) reveals a rough correspondence to their geographic location: cities on the West Coast (with smaller values of $k$) are further to the left on Fig. 2 than those on the East Coast (with larger values of $k$). Furthermore, high-latitude cities generally experience larger values of $\Delta \,Water\,Level$ and thus require greater amounts of sea-level rise to induce a flooding regime shift, owing to the general increase in $\sigma$ with latitude.

The exponential growth of the odds of exceedance with sea-level rise appears slightly jagged (Fig. 4A,B) because these (empirical) distributions arise from observations and, thus, are not smooth in contrast to curves resulting from theoretical distribution such as GEV (see Extended Data Fig. 2). Unsurprisingly, we find that the low-latitude stations belonging to the red cluster are highly susceptible to this doubling, with the most susceptible of these sites (e.g., Hawaii and the Caribbean) experiencing a doubling in exceedance probability with nearly every centimeter of sea-level rise. On the other hand, stations belonging to the blue cluster, which indicates a higher value of the GEV shape parameter, $k$, are much less susceptible, since they require larger amounts of sea-level rise (~10–25 cm) to double in frequency. The rate of growth in the odds of exceedance due to sea-level rise is well predicted by the doubling function based on Eq. (5), i.e., $O/{O}_{0}={2}^{\frac{{\mu }_{{\rm{SL}}}}{\tilde{\sigma }}}$. The thin dashed lines on Fig. 4B enclose areas that are within a factor of 4 of the relationship in Eq. (5), and growth rate curves at 90% of the sites fall within a factor of 4 of Eq. (5) over ten doubling periods (i.e., 210, which represents more than a thousand-fold increase in the odds). Although previous works have estimated the amount of sea-level rise associated with a single doubling event (e.g., Vitousek *et al*. (2017)^5^), we demonstrate here, for the first time, the near constancy of the amount of sea-level rise that doubles the odds of exceedance over several doubling events, which is characteristic of exponential growth. Slight deviations from the pure form of exponential growth (i.e., the thick, dashed line in Fig. 4B) exist across the sites used here. For example, stations with large values of $\mu$ and $\sigma$ (colored in green) appear to taper away from the theoretical result after about ten orders of magnitude. On the other hand, stations with small values of $\mu$ and $\sigma$ (colored in red), which are the most susceptible to sea-level rise, generally fit the theoretical result the best among the different groups. Finally, we note that the amount of sea-level rise causing a doubling in the odds of exceeding the present-day 50-year water-level event, $\tilde{\sigma }$, is closely related to the GEV scale parameter $\sigma$ (see Extended Data Fig. 5), particularly for stations with small and intermediate values of $\mu$ and $\sigma$, and near-zero values of $k$. However, $\sigma$ and $\tilde{\sigma }$ are not the same. Further, both $\sigma$ and $\tilde{\sigma }$, individually, represent relevant parameters to describe the nature of (present-day and future) extreme water level events (as discussed further in Extended Data Fig. 5).

The amount of sea-level rise that doubles the odds of the exceeding the present-day 50-year water-level event, $\tilde{\sigma }$, is somewhat dissimilar between the U.S. East and West Coasts (Fig. 5). The U.S. West Coast, in general, experiences much smaller values of $\tilde{\sigma }$, with a mean of approximately 5.3 cm. However, the mean of $\tilde{\sigma }$ on the U.S. East Coast is approximately 10.8 cm. The U.S. East Coast is punctuated with locations that exhibit very large values of $\tilde{\sigma }$ at tide stations exposed to tropical storms. The highly susceptible sites (e.g., the red cluster in Fig. 1 and Fig. 5, with small values of the GEV parameters $\mu$, $\sigma$, and $k$) are associated with small values (typically < 5 cm) of $\tilde{\sigma }$. On the other hand, the stations exposed to tropical storms (with large values of the GEV parameter $k$), which are shown in blue, typically experience much larger values (typically > 5 cm) of $\tilde{\sigma }$. The histogram in panel B is fairly skewed towards smaller values of $\tilde{\sigma }$: nearly two-thirds (65%) of all stations used here have values of $\tilde{\sigma }$ less than 10 cm.

As with the amount of sea-level rise (Fig. 4), an exponential relationship also exists between the doubling of the odds of exceedance and time (Fig. 6). For most sites, this relationship most closely parallels a trend corresponding to a doubling in the odds of flooding every 5 years (Figure 6B,D,F). Once more, low latitude stations (in red) have their odds of flooding increased much faster than higher latitude stations. The stations that show the lowest growth rates occur where sea level is projected to fall in the short term (e.g., black lines on Fig. 6) due to local uplift from continental rebound associated with the melting glaciers and Greenland (i.e., Glacio-isostatic adjustment). For these few stations, impacts due to sea-level rise are delayed until about 2050 or later. For the rest of the stations considered here, the odds of exceedance increase by a factor of ~100 or more by 2050 for high-end sea-level rise scenarios. For the most susceptible sites, shown in red, even the lower RCP 2.6 emissions scenarios provide only a brief respite from the impacts of sea-level rise. The RCP 2.6 scenario sea-level rise projections are more than enough to double the flood frequency several times over at the most susceptible sites. Impacts occurring at the higher-latitude stations (shown in green) and the tropical-storm exposed stations (shown in blue), on the other hand, do seem to be delayed by a few decades under the RCP 2.6 sea-level rise scenario compared with the RCP 8.5 scenario.

Unlike $\tilde{\sigma }$, which demonstrates spatial inconsistency between the U.S. West and East Coasts, the timescale over which sea-level rise doubles the odds of exceeding the present-day 50-yr water level event, $\tau$, is surprisingly consistent across sites, particularly for the period 2025–2075 (Fig. 7D), which is likely due to the high rates of relative sea-level rise on the U.S. East Coast due to land subsidence. Like $\tilde{\sigma }$, the magnitude of $\tau$ at highly susceptible sites are much shorter ($\tau \le \, \sim \,5$ years) than the other sites (Fig. 7B,D). Within the 2025–2075 time period, $\tau$ is centered on 5 years: 70% of tides stations used here have values of the doubling time scale greater than 4 years and less than 6 years. 91% of all stations used here have values of $\tau$ < 7 years for the period 2025–2075.

Finally, we discuss the finding of the fairly consistent 5-year time scale to double the odds of exceeding the 50-year extreme water level. The amount of sea-level rise that doubles the odds of exceedance (with units of length), $\tilde{\sigma }$, and the time scale that doubles the odds of exceedance (with units of time), $\tau$, are related by the rate of sea-level rise (with units of length per time). Based on the sea-level rise projections of Kopp *et al*., (2014)^29^, the average rate of sea-level rise over the period of 2025–2075 is 1.68 cm/year for RCP 8.5. Simply dividing the mean value of  $\tilde{\sigma }$, 9.2 cm, by this average rate of sea-level rise results in a time scale of 5.5 years. Hence, we have simplified our understanding of the relative impacts of sea-level rise at a given site to an interaction between three variables: the present-day variance of extreme water levels, the amount that sea-level rise modifies that extreme water-level variance, and the time scales over which this modification occurs due to sea-level rise projections. Although coastal water-level hazards vary across a multitude of spatiotemporal scales, nonlinear processes, and interactions, the consequences of sea-level rise alone on a stationary extreme water-level climate are profound, even without considering potential changes in storminess.

## Conclusions

The long-term trend in mean sea level has profound consequences on the nature of extreme events. Present-day extreme water-level events will become commonplace within the next few decades. Given established emissions trajectories and sea-level projections, the odds of extreme coastal flooding will double every 5 years into the foreseeable future at most locations in the U.S. The near-constancy (in space and time) of the 5-year doubling period found here is particularly consequential: Sea-level rise will likely increase the odds of flooding by a thousand-fold (i.e., 210) in a half-century. By 2100, today’s ‘once-in-a-lifetime’ (e.g., 50-year return period) coastal flood level may be exceeded every day during the highest tide at over 90% of our 202 considered U.S. sites. With increased flood frequency, we expect a corresponding acceleration of a number of related coastal hazards, such as beach and cliff erosion^49–51^. Our society has yet to fully comprehend the imminence of the projected regime shifts in coastal hazards and the consequences thereof.

## Methods

### Extreme value theory

Following the well-known frequentist interpretation of extreme statistics, we circumvent the temporal dependence of extreme events by considering the exceedance probability distribution, which is given by

1 $$E(x)=1-F(x),$$

where $F(x)$ is the cumulative probability distribution and $x$ is the magnitude of a random variable, e.g., extreme water level. Exceedance distributions are monotonically decreasing functions ranging from 1 to 0 with increasing $x$. When $x$ represents the extremes of a random variable, the probability/exceedance distributions are typically characterized using the Generalized Extreme Value (GEV) or the Generalized Pareto Distribution (GPD) statistical models^32,52–54^, as discussed below. As the mean of the exceedance distribution is increased, in this case due to sea-level rise, the probability of an event exceeding a particular threshold increases relative to the previous state. Since the distribution’s mean increases with time (e.g., due to the time-dependence of sea-level rise projections), so too does the exceedance probability increase with time. Yet, in taking this so-called “stationary” approach, we have isolated the *rate* of increase with time due to sea-level rise from the multitude of dynamic, yet transient sea-level fluctuations, which may cause “non-stationary” (e.g., seasonal and long-term) changes to the underlying exceedance distribution.

Many probability distributions exhibit an exponential decay in probability with the magnitude of the random variable^55^. Hence, a shifting baseline (or increased mean) of the random variable results in an exponential increase in the probability of exceeding a given threshold. By applying the Gumbel distribution with a shifting mean due to sea-level rise, Hunter (2012)^15^ found that the number of extreme events exceeding a particular threshold increases exponentially with sea-level rise, given by

2 $$\frac{N}{{N}_{0}}=\exp \left(\frac{{\mu }_{{\rm{SL}}}}{\sigma }\right),$$

where $N$ is the number of expected events under higher sea-level rise, ${N}_{0}$ is the current number of events without sea-level rise, ${\mu }_{{\rm{SL}}}$ is the sea level increase, and $\sigma$ is the scale parameter of the Gumbel distribution. Eq. (2) is modified when accounting for the family type of the extreme value distribution^42^. Here, we further explore sea level’s role in increasing the frequency of flooding events by focusing on a different form of exponential growth. We investigate an exponential function of the form

3 $$\frac{E}{{E}_{0}}={2}^{\frac{{\mu }_{{\rm{SL}}}}{\tilde{\sigma }}},$$

where $E$ is the exceedance probability distribution of extreme water level under future sea-level conditions, and ${E}_{0}$ is the exceedance probability distribution in present-day conditions (see Extended Data Figs. 1 and 3). In the present study, we focus on the exceedance probabilities of the 50-year water-level event. The ratios of the exceedance probability in Eq. (3) represents the commonly used ‘multiplying factors’^15^, ‘amplification factors’^16^, or ‘factors of increase’^5^, as discussed below. In Eq. (3), ${\mu }_{{\rm{SL}}}$ is once again the sea-level increase, and $\tilde{\sigma }$ is the amount of sea-level rise needed to double the exceedance probability. $\tilde{\sigma }$ is comparable to the GEV/GPD scale parameter, $\sigma$, but the two variables are not the same due to the influence of the GEV/GPD family-type parameter, $k$, as discussed below and in Extended Data Fig. 5.

We suggest that the functional form ${2}^{x}$ is slightly more intuitive than $\exp (x)$, since the argument $x$ in the function ${2}^{x}$ clearly indicates the number of doubling events. For example, when $\tilde{\sigma }$ is found to be 5 cm, then 5 cm of sea-level rise (${\mu }_{{\rm{SL}}}=5$ cm; ${\mu }_{{\rm{SL}}}/\tilde{\sigma }=1$) doubles the exceedance probability corresponding to a particular threshold. An additional 5 cm of sea-level rise (${\mu }_{{\rm{SL}}}=10$ cm; ${\mu }_{{\rm{SL}}}/\tilde{\sigma }=2$) doubles the exceedance probability again. Vitousek *et al*. (2017)^5^ found global-scale estimates of the amount of sea-level rise needed for a single frequency doubling to be around 5–10 cm, with lower-end values needed in the tropics. They estimated the amount of sea-level rise required for a single frequency doubling by estimating the difference in water level between the 50-year and 25-year events. Here, we examine the continuous process of doubling via the relationship between $E/{E}_{0}={E}_{0}({x}_{50}-{\mu }_{{\rm{SL}}})/{E}_{0}({x}_{50})$ and ${\mu }_{{\rm{SL}}}$, where ${x}_{50}$ is the magnitude of the 50-year water level event. Note that ${E}_{0}(x-{\mu }_{{\rm{SL}}})$ simply represents a translation of the initial exceedance probability distribution by the amount ${\mu }_{{\rm{SL}}}$ as illustrated in Fig. 8. For this relationship, ${\tilde{\sigma }}^{-1}$ is the slope of the relationship between ${\log }_{2}(E/{E}_{0})$ and ${\mu }_{{\rm{SL}}}$. In the equations given above, the exceedance distribution $E(x)$ can be estimated from a variety of methods and models. For example, $E(x)$ can be obtained empirically from data or can be fit to a popular extreme value model such as GEV or GPD, as described below. The equations given here are agnostic to the underlying form of $E(x)$.

In addition to estimating the increase in the exceedance probability, we also examine the rate of increase in the odds of occurrence, $O(x)$, which is defined as

4 $$O(x)=\frac{E(x)}{1-E(x)}.$$

The odds of occurrence in Eq. (4), represents the ratio of the probability of an event exceeding a particular threshold to the probability of an event NOT exceeding the threshold. Likewise, we examine the exponential growth rate in the odds of exceedance, which is given by

5 $$\frac{O}{{O}_{0}}={2}^{\frac{{\mu }_{{\rm{SL}}}}{\tilde{\sigma }}}.$$

We consider the analysis of Eq. (5) to be preferable to Eq. (3) because the factor of increase in the odds, i.e., *O*/*O*_0_, is unbounded, whereas $E/{E}_{0}$ is not. The factor of increase in exceedance probability, $E/{E}_{0}$, exhibits an upper bound since $E$ has an upper bound of 1. The odds, *O*, on the other hand, is unbounded. The unbounded nature of Eq. (5) renders it valid for a much larger range of sea-level rise, i.e., ${\mu }_{{\rm{SL}}}$, compared to that for Eq. (3). For example, Eq. (3) might only be valid for the first five or six ‘doubling’ events (as seen in Extended Data Figs. 1 and 2), whereas the Eq. (5) appears valid for at least ten ‘doubling’ events for the 90% of the tide stations used here, as discussed in the Results section pertaining to Fig. 4. The appearance of upper bounds on the factor of increase, $E/{E}_{0}$, in the current analysis are discussed further in Extended Data Figs. 1 and 3. A schematic showing the exceedance probability and the odds of increase of the GEV distribution are shown in Fig. 8, which illustrates the current methodology to calculate $E/{E}_{0}$ and *O*/*O*_0_. Figure 8A,B also motivate our consideration of the exponential rates of growth described in Eqs. (3) and (5), respectively.

Because sea-level rise is a function of time and space, we investigate a slight modification of Eq. (5), given by

6 $$\frac{O}{{O}_{0}}={2}^{\frac{t}{\tau }},$$

where $t$ is time and $\tau$ represents the time scale for doubling. The results in Figs. 6 and 7, apply Eq. (6) for the doubling time $\tau$ at individual tide stations subject to the site-specific sea-level rise curves of Kopp *et al*. (2014)^29^.

### Generalized Extreme Value (GEV) distribution

The cumulative probability function of the Generalized Extreme Value (GEV) distribution is given by

7 $$F(x;\mu ,\sigma ,k)={e}^{-{\left(1+k\left(\frac{x-\mu }{\sigma }\right)\right)}^{-1/k}},$$

where $x$ is a random, independent variable (in this case, $x$ represents the set of $r=3$ independent annual maxima observations of the hourly water level as discussed below in Application), ${e}^{x}=\exp (x)$ is the exponential function, and $\mu$, $\sigma$, and $k$ represent the location, scale, and shape parameters, respectively^32^. The location, scale, and shape parameters represent proxies for the distributions mean, standard deviation (e.g., width), and tail behavior. Depending on the sign of the shape parameter, $k$, the function $F(x)$ may exhibit an upper bound (i.e., when $k < 0$) or a lower bound (i.e., when $k > 0$). The GEV distribution generalizes three families of extreme value distributions: Gumbel (type I), Fréchet (type II) and Weibull (type III), corresponding to values of the shape parameter $k=0$, $k > 0$, and $k < 0$, respectively.

From Eq. (7), the distribution of the exceedance probability, i.e., the probability that a given water level $x$ is exceeded, is given by

8 $$E(x;\mu ,\sigma ,k)=1-F=1-{e}^{-{\left(1+k\left(\frac{x-\mu }{\sigma }\right)\right)}^{-1/k}}.$$

The factor of increase in exceedance probability of an elevated sea-level condition (${\mu }_{{\rm{SL}}} > 0$) relative to a neutral baseline (${\mu }_{{\rm{SL}}}=0$) is thus given by

9 $${f}_{{\rm{inc}}}(x;\mu ,{\mu }_{{\rm{SL}}},\sigma ,k)=\frac{E(x;\mu +{\mu }_{{\rm{SL}}},\sigma ,k)}{E(x;\mu ,\sigma ,k)}=\frac{1-{e}^{-{(1+k(\frac{x-\mu -{\mu }_{{\rm{SL}}}}{\sigma }))}^{-1/k}}}{1-{e}^{-{(1+k(\frac{x-\mu }{\sigma }))}^{-1/k}}}$$

Although Eq. (9) is exact for known parameters, it is unwieldy. We simplify Eq. (9) to an approximate form that illuminates the critical role of the sea-level rise parameter, ${\mu }_{{\rm{SL}}}$. After approximating the exponential functions in Eq. (9) using Taylor series, ${e}^{s}=1+s+\frac{{s}^{2}}{2!}+\frac{{s}^{3}}{3!}+\mathrm{..}.$, and retaining only the first two terms in the expansion, the factor of increase in exceedance probability becomes

10 $${\bar{f}}_{{\rm{inc}}}(x;\mu ,{\mu }_{{\rm{SL}}},\sigma ,k)=\frac{{\left(1,+,k,(,\frac{x-\mu }{\sigma },)\right)}^{1/k}}{{\left(1,+,k,(,\frac{x-\mu -{\mu }_{{\rm{SL}}}}{\sigma },)\right)}^{1/k}}$$

The Taylor series approximation is valid for small values of the arguments of the exponential function, $s=-\,{\left(1+k\left(\frac{x-\mu -{\mu }_{{\rm{SL}}}}{\sigma }\right)\right)}^{-1/k}$ or $s=-\,{\left(1+k\left(\frac{x-\mu }{\sigma }\right)\right)}^{-1/k}$, in Eq. (9), which correspond to large event levels, $x$. Letting the shape parameter $k\to 0$, Eq. (10) becomes

11 $${\tilde{f}}_{{\rm{inc}}}=\mathop{\mathrm{lim}}\limits_{k\to 0}\,{\bar{f}}_{{\rm{inc}}}(x;\mu ,{\mu }_{{\rm{SL}}},\sigma ,k)=\frac{\mathop{\mathrm{lim}}\limits_{k\to 0}{\left(1,+,k,(,\frac{x-\mu }{\sigma },)\right)}^{1/k}}{\mathop{\mathrm{lim}}\limits_{k\to 0}{\left(1,+,k,(,\frac{x-\mu -{\mu }_{{\rm{SL}}}}{\sigma },)\right)}^{1/k}}=\frac{{e}^{\frac{x-\mu }{\sigma }}}{{e}^{\frac{x-\mu -{\mu }_{{\rm{SL}}}}{\sigma }}}={e}^{\frac{{\mu }_{{\rm{SL}}}}{\sigma }},$$

since $$\mathop{\mathrm{lim}}\limits_{n\to \infty }{\left(1+\frac{s}{n}\right)}^{n}=\mathop{\mathrm{lim}}\limits_{k\to 0}{(1+ks)}^{1/k}={e}^{s}$$ with $$n=\frac{1}{k}$$.

Eq. (11) shows that, to a good approximation, the probability of flooding increases exponentially with sea-level rise with an exponent inversely proportional to scale parameter, $\sigma$. Notably, the factor of increase is independent of $x$. However, the Taylor series expansion and limit as $k\to 0$, have the effect of making the approximation (11) most accurate for large events $x$ and small absolute values of  $k$.

Applying the Gumbel distribution (i.e., $k=0$), Hunter (2012)^15^ found that the expected number of exceedance events, $N$, increases exponentially with sea-level rise relative to $\sigma$ according to Eq. (11). Here, we demonstrate the relevance of that result to understand both the probability of exceedance and the behavior of the GEV distribution. For the majority of the cases where $k < 0$ (e.g., 82% of the stations used in the current study), Eq. (11) represents a lower bound to the growth rate. Nevertheless, the simplified formulation, Eq. (11), provides important insights into the expected growth rates of flood frequency.

An increase in the exceedance probability of extreme events is equivalent to a decrease in the return period, ${T}_{R}$, given by

12 $${T}_{R}=\frac{{r}_{i}}{E(x)},$$

in which ${r}_{i}$ is the recurrence interval from the observed data. For example, a 100-year event has an exceedance probability of 0.01, that is, a 1% chance of occurring in a given year (with ${r}_{i}=1$ year, i.e., a distribution fit to the annual maxima). The return period concept is often more intuitive than the probability of rare events. We can estimate the factor of decrease in return period at the new sea-level state (${\mu }_{{\rm{SL}}} > 0$) relative to the neutral baseline (${\mu }_{{\rm{SL}}}=0$) as

13 $$\frac{{T}_{R}(x;\mu +{\mu }_{{\rm{SL}}},\sigma ,k)}{{T}_{R}(x;\mu ,\sigma ,k)}={\left[\frac{E(x;\mu +{\mu }_{{\rm{SL}}},\sigma ,k)}{E(x;\mu ,\sigma ,k)}\right]}^{-1}={f}_{{\rm{inc}}}^{-1}\approx {\bar{f}}_{{\rm{inc}}}^{-1}\mathop{\to }\limits^{k\to 0}{\tilde{f}}_{{\rm{inc}}}^{-1}={e}^{-\frac{{\mu }_{{\rm{SL}}}}{\sigma }}.$$

Eq. (13) demonstrates that an increase in exceedance probability corresponds to a decrease in the return period that is governed by the inverse of Eq. (9), and approximately by the inverse of Eq. (11). Thus, an exponential increase in flooding frequency or exceedance probability corresponds to an exponential decay in return period at that event level.

### Generalized Pareto distribution (GPD)

We show that the exponential form of increase remains valid for the generalized Pareto distribution (GPD), i.e., the probability distribution of values exceeding a threshold or, likewise, the distribution of the asymptotic tail of a random variable^53,54^. The cumulative probability function of the Generalized Pareto distribution is given by

14 $$F(x;\mu ,\sigma ,k)=1-{\left(1,+,k,(,\frac{x-\mu }{\sigma },)\right)}^{-1/k},$$

where $x$ is a random, independent variable representing the water level and $\mu$, $\sigma$, and $k$ represent the location, scale, and shape parameters, respectively^32^. The exceedance probability distribution of the GPD is given by

15 $$E(x;\mu ,\sigma ,k)={\left(1,+,k,(,\frac{x-\mu }{\sigma },)\right)}^{-1/k}.$$

Thus, the factor of increase in exceedance probability of a new sea-level condition (${\mu }_{{\rm{SL}}} > 0$) relative to a neutral baseline (${\mu }_{{\rm{SL}}}=0$) is given by

16 $${f}_{{\rm{inc}}}(x;\mu ,{\mu }_{{\rm{SL}}},\sigma ,k)=\frac{E(x;\mu +{\mu }_{{\rm{SL}}},\sigma ,k)}{E(x;\mu ,\sigma ,k)}=\frac{{\left(1,+,k,(,\frac{x-\mu }{\sigma },)\right)}^{1/k}}{{\left(1,+,k,(,\frac{x-\mu -{\mu }_{{\rm{SL}}}}{\sigma },)\right)}^{1/k}}={\bar{f}}_{{\rm{inc}}}.$$

Note that the *unapproximated* result for the GPD given in Eq. (16) is identical to the *approximated* result (i.e., using Taylor series approximation) of the GEV increase function ${\bar{f}}_{{\rm{inc}}}$, Eq. (10). Interestingly, this behavior might be expected because the GPD, Eq. (14), represents the first two terms in a Taylor series expansion of the GEV distribution (7). Furthermore, this behavior is consistent with the theory that the GPD represents the tails of another distribution and that retaining the first two terms in a Taylor series expansion renders the distribution valid for the largest event levels $x$, which comprise the tails of the GEV distribution. Also as expected, in the limit that  $k\to 0$, Eq. (16) becomes Eq. (11).

### Number of exceedances

Hunter (2012)^15^ investigates the increase in the frequency of extreme water-level events using the expected number of exceedances, $N$, rather than the increase in exceedance probability (or decrease in return period).

The number of exceedances, $N$, is related to the cumulative probability distribution, $F$ by

17 $$F={e}^{-N}.$$

Based on the GEV cumulative probability distribution (given in Eq. (7)), Eq. (17) can be expressed as

18 $$N(x;\mu ,\sigma ,k)=\frac{1}{{\left(1,+,k,(,\frac{x-\mu }{\sigma },)\right)}^{1/k}}.$$

In the limit that the shape parameter $k\to 0$, Eq. (18) becomes

19 $$\bar{N}(x;\mu ,\sigma ,k)={e}^{-\frac{x-\mu }{\sigma }}.$$

Eq. (19) is identical to previous formulations in Hunter (2012)^15^, who considered the Gumbel form $(k=0)$ of the GEV distribution. Based on Eq. (19), the factor of increase in the number of exceedances as a function of sea-level rise can be written as

20 $${N}_{{\rm{inc}}}=\frac{\bar{N}(x;\mu +{\mu }_{{\rm{SL}}},\sigma ,k)}{\bar{N}(x;\mu ,\sigma ,k)}={\tilde{f}}_{{\rm{inc}}}={e}^{\frac{{\mu }_{{\rm{SL}}}}{\sigma }}.$$

Eq. (20) demonstrates that the increase in the number of exceedances as a function of sea-level rise mimics the approximate form of the increase in exceedance probability, given by Eq. (11). The number of exceedances, Eq. (20), may be preferable to the exceedance probability, Eq. (11), or return period, Eq. (13), because, like the GPD, the Taylor series approximation is not required to produce the simplified exponential expression. However, to obtain the purely exponential behavior we again require $k\to 0$. Nevertheless, there appears to be strong theoretical grounds indicating an exponential increase in both the probability and/or number of exceedance events associated with a baseline trend in the random variable. In the theoretical results discussed above, the natural exponential function ($\exp (x)$) clearly plays a critical role. Yet, in practice, we have demonstrated that alternate exponential forms, e.g., ${2}^{x}$, can accurately assess the increases in the frequency of extreme events, using an intuitive relationship.

### Application

The National Oceanic and Atmospheric Association (NOAA) archives water-level observations along the U.S. coast via the Tides and Currents database (https://tidesandcurrents.noaa.gov/). We obtained the data used in the current study from the NOAA CO-OPS Application Programming Interface (API), described here: https://tidesandcurrents.noaa.gov/api/. Using the API, we downloaded hourly sea-level records from 1950–2017 for 876 stations around the U.S. As is common practice in extreme value analysis, we removed any linear trend in the hourly water-level observations in order to eliminate the influence of sea-level rise on the magnitude and frequency of extreme events (see e.g., Tebaldi *et al*., (2012)^17^). We restricted our study to only use stations whose records contain at least ten years of sea-level observations. This restriction ensures sufficient data quantity for parameter estimation of the extreme value distribution. For the remaining stations, we identified the top 10 maximum sea-level events with a minimum peak separation of at least three days to ensure that the block maxima are independent. Later, we applied only the top three annual maxima ($r=3$) to obtain GEV parameter estimates, leaving the remaining 4^th^ -10^th^ largest events to fill in any data gaps, if present. The choice of fitting a GEV distribution to the $r=3$ annual maxima instead of the $r=1$ (largest event per year) is a modification of the $r$-largest order statistic model for block maxima described in Coles *et al*., (2001)^32^. Unlike in Coles *et al*. (2001)^32^, we do not assess the limiting distributions associated with different values of $r$, and instead simply fit a GEV distribution (using maximum likelihood estimates) to the $r=3$ annual maxima since estimates of extreme event magnitude begin to stabilize around $r\ge 3$ (see e.g., Vitousek & Fletcher (2008)^56^). Selecting $r=3$, the recurrence interval ${r}_{i}$ in Eq. (12) is given by $1/r=1/3$ years. We chose to select the top three annual maxima in order to decrease the influence of large, isolated water-level events, such as those resulting from tropical storms. Additionally, we removed outliers in the top three annual maxima, identified as events whose magnitude exceeded three median absolute deviations (MADs) of the set block maxima^57^. The outlier removal procedure typically (i.e., 55% of the time) removed three or fewer outliers from the set of the top 3 annual maxima for the entire water-level record. We also note that the removal of the outliers only affects the characterization of the GEV distribution and the estimation of the 1-year and 50-year return water levels. For the growth rate analysis (shown in Figs. 4–8), we use the full empirical distribution (i.e., without removing any outliers). Gaps in the block maxima, which were created by removing outliers or that occurred from year-long gaps in the observation time series, were filled with the largest remaining events of the sorted data from the set of the remaining 4^th^ -10^th^ largest events from all years. If the station lacked sufficient data from the (4^th^ -10^th^ largest) block maxima of recorded years to fill in prolonged gaps in the observations, then the station was eliminated from the current analysis. However, this case represented only 4% of the stations eliminated here. On the other hand, we removed 66% of the stations because the data record was less than 10 years. Of the remaining stations, the average record duration was approximately 37 years (minimum duration = 10 years; maximum duration = 68 years [i.e., 1950–2017]). Another 5% of stations that did not record water-level observations at hourly time scales were eliminated. The data quality control procedure thus retained nearly 25% of all of the stations (i.e., 217 of stations out of the initial 876 stations). Yet, we further eliminated a small subset of the remaining stations that behaved as outliers in terms of the derived GEV parameters. We omit stations whose derived $\mu$ and $\sigma$ parameters exceed 3 MADs of the retained stations. However, we do not eliminate any stations based on the $k$ parameter of the GEV distribution, since $k$ is, in general, more variable than $\mu$ and $\sigma$, in part because $k$ can be either positive or negative (whereas both $\mu$ and $\sigma$ are positive). Furthermore, we retain the stations exhibiting extraordinarily large (absolute) values of $k$ to demonstrate that including these stations does not change the conclusions derived herein with respect to imminent transitions in flooding regimes. By eliminating stations with outlying values for $\mu$ and $\sigma$, only 202 stations remain in the present study (Fig. 1A).

We calculate the difference in extreme water level, $\Delta Water\,Level$, according to

21 $$\Delta W{L}_{50{\rm{yr}}\to 1{\rm{yr}}}=x({T}_{R}=50;\mu ,\sigma ,k)-x({T}_{R}=1;\mu ,\sigma ,k),$$

which is simply the difference between the 50-yr water level, $x({T}_{R}=50;\mu ,\sigma ,k)$, and the 1-yr water level, $x({T}_{R}=1;\mu ,\sigma ,k)$, of the unaltered GEV water-level distribution (left panel of Fig. 2). Here, the function $x({T}_{R};\mu ,\sigma ,k)$ is obtained by inverting the GEV exceedance distribution, $E(x)$, to find the value of the random variable, $x$, corresponding to an exceedance probability of $E=\frac{{r}_{i}}{{T}_{R}}$. Additionally, we calculate the other $\Delta Water\,Level$ scenario (right panel of Fig. 2), according to

22 $$\Delta W{L}_{50{\rm{yr}}\to {\rm{MHHW}}}=x({T}_{R}=50;\mu ,\sigma ,k)-{\rm{MHHW}},$$

where $x({T}_{R}=50;\mu ,\sigma ,k)$ is 50-yr water level and Mean Higher High Water (MHHW), which is calculated as the average of the larger semidiurnal high tide events from the detrended time series of the hourly water-level observations at each station. To obtain the year in the future when the $\Delta Water\,Level$ scenarios (shown in Fig. 2) are exceeded by the sea-level rise projections of Kopp *et al*. (2014)^29^, we apply a linear interpolation of the time series $t$ vs. sea-level rise to find the abscissa (i.e., time $t$) associated with an ordinate equal to $\Delta Water\,Level$. The error bars on Fig. 3 represent the 95% confidence levels based on the probabilistic sea-level rise projections of Kopp *et al*. (2014)^29^. Specifically, the lower bounds of the error bars in Fig. 3 represent the upper end of the sea-level rise projections (i.e., the 97.5% quantile) and, likewise, the upper bounds of the error bars represent the lower end of the sea-level rise projections (i.e., the 2.5% quantile). Hence, 95% of the sea-level rise projections fall between the error bars shown in Fig. 3.

Figure 4 shows the odds increase calculated from three different distributions of exceedance probability. The relative odds is calculated from the equation

23 $$O/{O}_{0}={O}_{0}({x}_{50}-{\mu }_{{\rm{SL}}})/{O}_{0}({x}_{50}),$$

where  ${O}_{0}(x)=\frac{E(x)}{1-E(x)}$ is the odds of exceedance at present-day sea level, ${\mu }_{{\rm{SL}}}$ is the projected future sea-level, and ${x}_{50}$ is the magnitude of the 50-year water-level event. In the case of the GEV model shown in Extended Data Fig. 1C, this can be evaluated analytically via a shift in the mean of the distribution according to Eq. (8). For Figure 4, which is based upon the empirical exceedance probability distributions, we obtain the relative odds increase via linear interpolation.

In Fig. 6, we again remap the abscissa in Fig. 4 from sea-level rise to time using linear interpolation and the sea-level rise projections of Kopp *et al*. (2014)^29^, as we did in Fig. 3 (as discussed above). Here, we apply three different sea-level rise projections based on greenhouse gas concentration trajectory scenarios (RCP 2.6, RCP 4.5, and RCP 8.5), which correspond to a range of possible radiative forcing conditions in the year 2100.

## Supplementary information

Extended Data.

Dataset 1.
