# Supplementary material for: Sea-level rise exponentially increases coastal flood frequency
Source: Sci Rep. 2020 Apr 16;10:6466. doi: 10.1038/s41598-020-62188-4 (PMC7162943; doi:10.1038/s41598-020-62188-4)
Supplement: Supplementary file 1 — Extended Data. [file 41598_2020_62188_MOESM1_ESM.docx]

**Sea-level rise exponentially increases coastal flood frequency**

Mohsen Taherkhani^a^, Sean Vitousek^a,b,*^, Patrick L. Barnard^b^, Neil Frazer^c^, Tiffany R. Anderson^c^, Charles Fletcher^c^

^a^University of Illinois at Chicago, Department of Civil & Materials Engineering, Chicago, IL, 60607, USA

^b^United States Geological Survey, Pacific Coastal and Marine Science Center, Santa Cruz, CA, 95060, USA

^c^University of Hawai‘i at Mānoa, School of Ocean and Earth Science and Technology, Department of Earth Sciences, Honolulu, HI, 96822, USA

*corresponding author: [svitousek@usgs.gov](mailto:vitousek@usgs.gov)


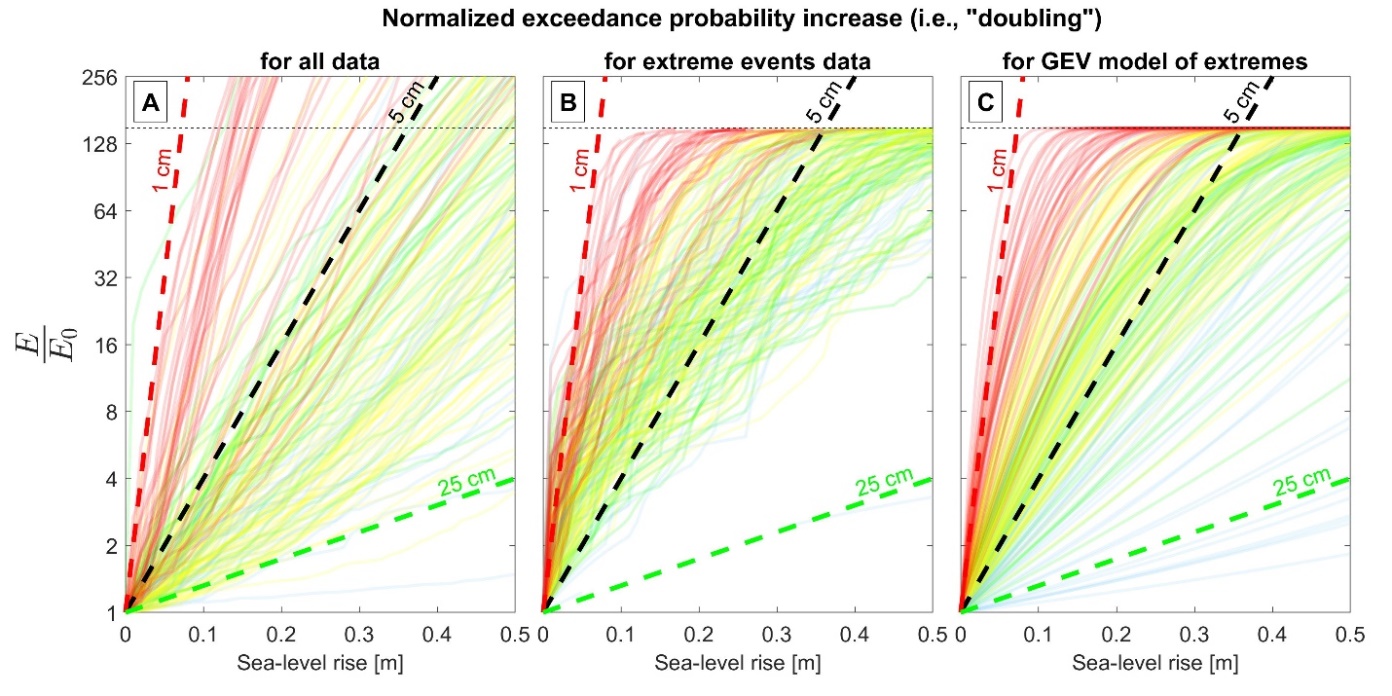


Extended Data Figure 1 – The relationship between SLR and the relative increase in the probability of exceeding the present-day 50-year water-level event, $E/E_{0}$, where, $E_{0}\left(x\right)$, is the present-day exceedance probability distribution and $E=E_{0}\left(x-\mu_{\text{SL}}\right)$ is the future (shifted) empirical exceedance probability distribution, where $\mu_{\text{SL}}$ represents the amount of future SLR. We calculate the present-day exceedance distribution, $E_{0}\left(x\right)$, in three different ways: panel A applies the empirical exceedance probability distribution, $E_{0}\left(x\right)$, resulting from $x$ being all values of recorded hourly water level; Panels B and C, on the other hand, apply (extreme) exceedance probability distributions, $E_{0}\left(x\right)$, resulting from $x$ being the top three annual maxima. Panel B uses the empirical exceedance distribution of the extremes, whereas panel C uses a best-fit GEV model for the exceedance distribution of the extremes. The x- and y-axes of the current figure are on linear and (base-two) logarithmic scales, respectively. Hence, relationships that follow a straight line correspond to exponential growth with SLR on the x-axis. Each solid line corresponds to the relationship between $E/E_{0}$ and SLR for a single tide station, and each station is colored according to its classification shown in Figure 1. The red, black, and green dashed lines correspond to a doubling of exceedance probability with every 1 cm, 5 cm, and 25 cm of SLR, respectively, according to Eq. (3).

Extended Data Figure 1 illustrates the relationship between SLR and the relative increase in the probability of exceeding the present-day 50-year water-level event, $E/E_{0}$. The relative probability increase is equivalent to a time-dependent ‘amplification factor’ or ‘factor of increase’, as investigated previously in Buchanan et al. (2017)^16^ or Vitousek et al. (2017)^5^, respectively, corresponding to the 50-year event. We calculate the future exceedance probability, $E\left(x\right)$, (where $x$ represents the water level) by shifting the present-day probability distribution $E_{0}$, by a variable amount of SLR. Thus, the future exceedance probability is given by $E=E_{0}\left(x-\mu_{\text{SL}}\right)$, which corresponds to increasing the mean value, μ, of the distribution by an amount of SLR equal to $\mu_{\text{SL}}$. Thus, the quantity $E/E_{0}$ is the ratio of the future exceedance probability, $E$, at the present day 50-year water-level threshold and the old exceedance probability, $E_{0}$. Extended Data Figure 1 shows the results for three different distributions of exceedance probability. Panel A applies the empirical exceedance probability distribution, $E_{0}\left(x\right)$, resulting from $x$ being all values of the recorded hourly water level. Panels B and C, on the other hand, apply exceedance probability distributions resulting from the top three annual maxima. Panel B uses the empirical exceedance distribution of extremes, whereas panel C uses the GEV model for the exceedance distribution of extremes. Unlike Figure 4A, the relative exceedance probability increases shown in Figure 4B and C are bounded within the axis limits. Since panels B and C are constructed from the exceedance probability distribution of extremes (i.e., top 3 annual maxima, $r_{i}=3$), the probability increase cannot exceed a factor of $E/E_{0}=1/E_{0}=1/\left(r_{i}/T_{r}\right)=1/\left(1/3\cdot1/50\right)=150$, despite the ever-increasing hazard level. Thus, considering the exceedance probability distribution of all hourly water levels (e.g., panel A) may be more informative to describe rates of growth than the probability distribution of extremes. In the case of panel A, $E_{0}=E_{0}\left(x=x_{50\text{ yr}}\right)$ corresponds to the probability of exceeding the water level established by the GEV distribution at the 50-year return period. Hence, $E_{0}$ based on the empirical (‘all values’) distribution is much smaller than the corresponding $E_{0}$ for the distributions of block maxima in panels B and C (since the empirical distribution is derived from vastly more data points). Therefore, upper bound on the empirically derived relative increase, $E/E_{0}$, is not encountered until much higher values of SLR (beyond the axis limits of Figure 4). Here, we apply three different representations of the exceedance distribution to demonstrate that the rates of growth are, for the most part, insensitive to the form of the exceedance distribution used. However, some differences exist among the three different distributions, which take a similar form to those for the relative odds increase, $O/O_{0}$, which are discussed below. In general, the relative odds increase, $O/O_{0}$, shown in Extended Data Figure 2, on the other hand, offer very similar rates of growth to Extended Data Figures 1, but are not bounded.


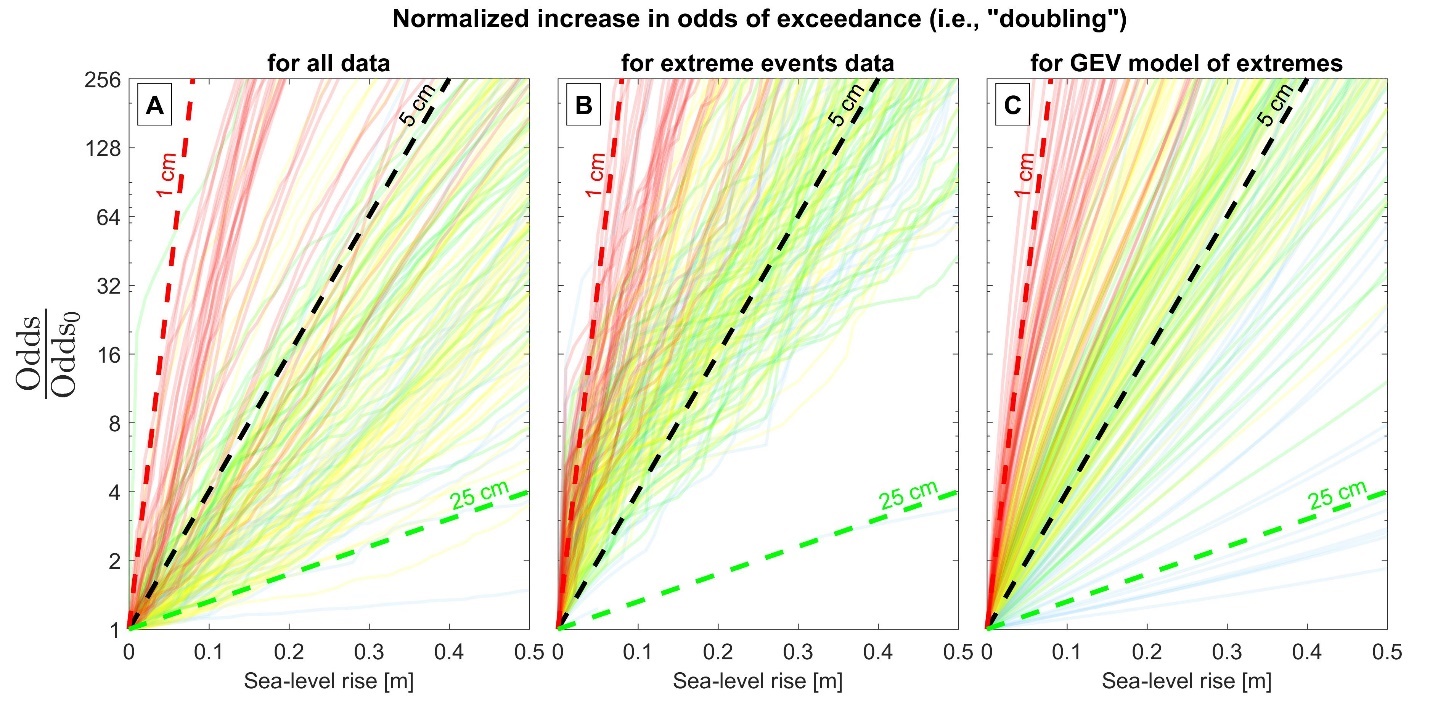


Extended Data Figure 2 – The relationship between SLR and the relative increase in the odds of exceeding the present-day 50-year water-level event, $O/O_{0}$, where, the odds are calculated as $O=E/\left(1-E\right)$ from the exceedance probability distribution $E$. As in Extended Data Figure 1, we apply the future (shifted) exceedance probability distribution as $E=E_{0}\left(x-\mu_{\text{SL}}\right)$, where $E_{0}\left(x\right)$ is the present-day exceedance probability distribution and $\mu_{\text{SL}}$ represents the amount of future SLR. We calculate the present-day exceedance distribution, $E_{0}\left(x\right)$, in three different ways: panel A applies the empirical exceedance probability distribution, $E_{0}\left(x\right)$, resulting from $x$ being all values of recorded hourly water level; Panels B and C, on the other hand, apply (extreme) exceedance probability distributions, $E_{0}\left(x\right)$, resulting from $x$ being the top three annual maxima. Panel B uses the empirical exceedance distribution of the extremes, whereas panel C uses a best-fit GEV model for the exceedance distribution of the extremes. The x- and y-axes of the current figure are on linear and (base-two) logarithmic scales, respectively. Hence, relationships that follow a straight line correspond to exponential growth with SLR on the x-axis. Each solid line corresponds to the relationship between $O/O_{0}$ and SLR for a single tide station, and each station is colored according to its classification shown in Figure 1. The red, black, and green dashed lines correspond to a doubling of exceedance probability with every 1 cm, 5 cm, and 25 cm of SLR, respectively, according to Eq. (3).

Extended Data Figure 2 illustrates the same relationships as in Extended Data Figure 1 but for the relative increase in the odds of exceeding the present-day 50-year water-level event, $O/O_{0}$ , due to SLR. The x- and y-axes of Extended Data Figures 1 and Extended Data Figure 2 are on linear and (base-two) logarithmic scales, respectively. Hence, relationships that follow a straight line correspond to exponential growth with SLR on the x-axis. Each solid line on Extended Data Figure 1 and Extended Data Figure 2 corresponds to the relationship between SLR and $E/E_{0}$ and $O/O_{0}$, respectively, for single tide station, which is colored according to its K-means cluster shown in Figure 1. The red, black, and green dashed lines correspond to a doubling of exceedance probability with every 1 cm, 5 cm, and 25 cm of SLR, respectively, according to Eq. (3). Hence, for vulnerable, low-latitude sites (shown in red), one centimeter of SLR can cause a doubling of the exceedance probability at the 50-year water level threshold, which is further explored in Discussion.

The curves on Extended Data Figure 1 and Extended Data Figure 2, calculated from the empirical exceedance probability distribution for all values of water level and for the extreme values of water level in panels A and B, respectively, are slightly jagged compared to panel C for the GEV distribution. The empirical distributions (shown in panels A and B) arise from the observed data and thus are not quite as smooth. In contrast, the GEV model represents a smooth fit to the observations. Although there are some notable differences between the three panels of Extended Data Figure 1 and Extended Data Figure 2, each panel shows roughly the same growth rates and the same variation among the growth rates for each cluster. In general, the curves on Extended Data Figure 1 and Extended Data Figure 2 parallel the dashed lines corresponding to doubling rates every 1 cm, 5 cm, and 25 cm of SLR arising from Eq. (5). Unsurprisingly, we find that the low-latitude stations belonging to the red cluster are highly vulnerable. The most vulnerable of these sites double in exceedance probability with nearly every centimeter of SLR. The curves of the most vulnerable sites (in red) derived from the extreme value distributions (panels B and C) appear slightly steeper than the ‘all-values’ distribution (panel A). Stations belonging to the blue cluster, which indicates a higher value of the GEV shape parameter, $k$, are much less vulnerable, since they require larger amounts of SLR (~10-25 cm) to double in frequency. Interestingly, the GEV modeled growth rates in panel C are larger for the most vulnerable sites (in red) and smaller for the least vulnerable sites (in green and blue) than the corresponding rates shown in panels A and B. Additionally, the empirical distribution of extremes in panel B exhibits less variability than in panels A and C. We offer that this is perhaps due to the limited variance among the observations of the extremes, compared with the variance of the all values distribution (panel A) or the variance of the smooth GEV model (panel C). In general, the smaller values of the variance will result in larger growth rates. Further, the empirical distributions of extremes (panel B) are less influenced by the presence of outliers (i.e., the very largest water level events), whereas the smooth, best-fit GEV models are highly dependent on the model fit to the tail of the distribution. Hence, there is a notable difference between the panels B and C for the curves with $k>0$, shown in blue. Despite the small differences between panels A, B, and C, on Extended Data Figure 1 and Extended Data Figure 2, there is a noticeable consistency overall among the growth rates using different forms of the exceedance distributions. Hence, the main body of the manuscript focuses on the empirical ‘all-values’ distribution as it does not rely on any procedure to categorize the extreme observations or any statistical model fit to those extremes. Further, we recommend the use of the ‘all-values’ distributions as it perhaps makes the most sense to illuminate the regime transition from “the extreme to the mean”^24^.


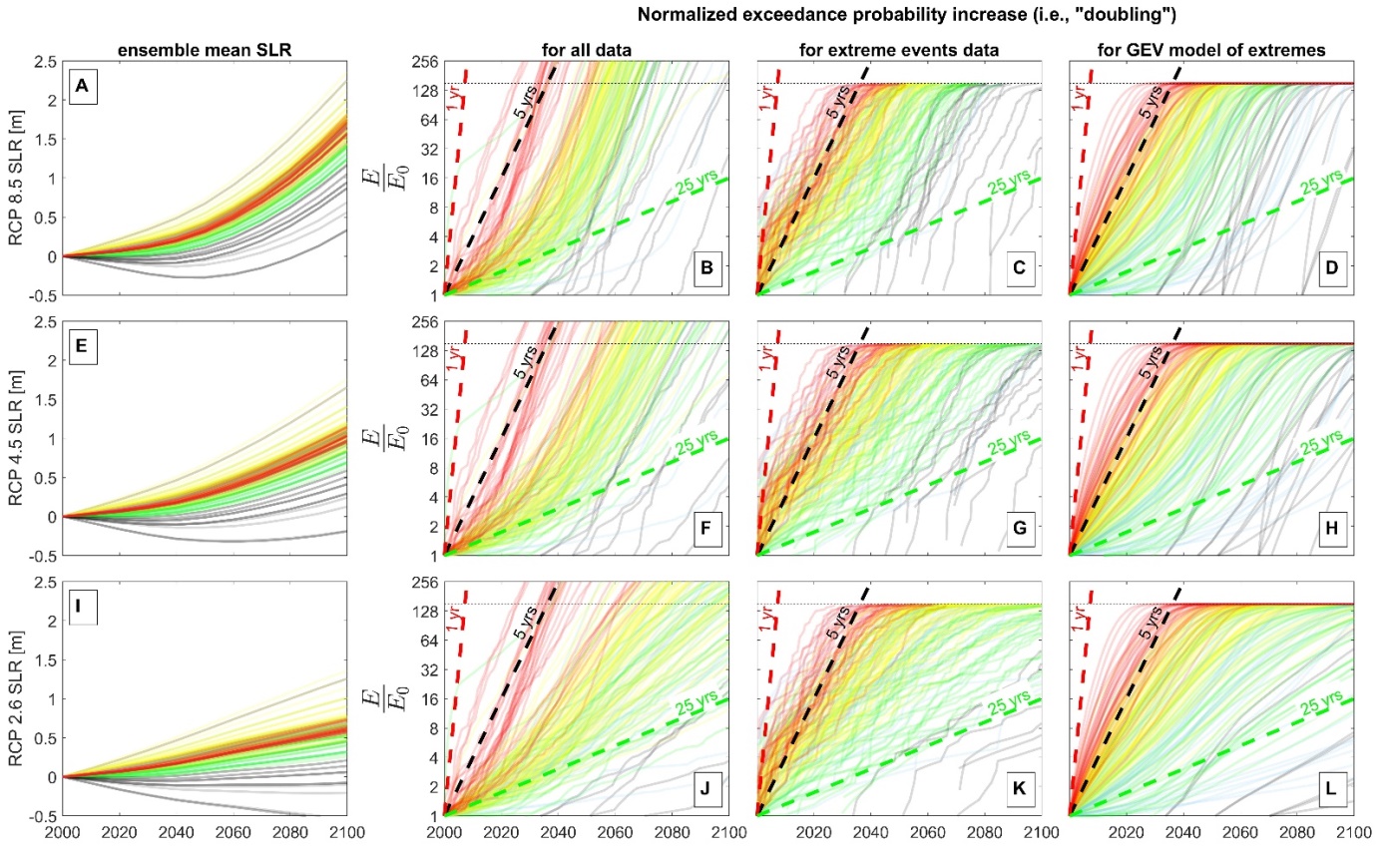


Extended Data Figure 3 - The relative increase in the probability of exceeding the present-day 50-year water-level event, $E/E_{0}$, with respect to time. As in Extended Data Figure 1, panels B, F, and J apply the empirical exceedance probability distribution resulting from all values of the recorded hourly water level. Panels C, G, and K apply the empirical exceedance distribution of the extremes, and panels D, H, and L use the best-fit GEV model for the exceedance distribution of the extremes. The x- and y-axes of the current figure are on linear and (base-two) logarithmic scales, respectively. Hence, relationships that follow a straight line correspond to exponential growth with time on the x-axis. Each solid line corresponds to the relationship between $E/E_{0}$ and SLR for a single tide station, and each station is colored according to its classification shown in Figure 1. The black solid lines correspond to tide stations where SLR is projected to decrease with time in the next few decades (which are located at high latitudes due to GIA). The red, black, and green dashed lines correspond to a doubling of exceedance probability with every 1 yr, 5 yrs, and 25 yrs, respectively, according to Eq. (3).

Extended Data Figure 3 illustrates the relative increase in the probability of exceeding the present-day 50-year water-level event, $E/E_{0}$, with respect to time for different SLR scenarios. The first column of Extended Data Figure 2 (i.e., panels A, E, and I) depicts the ensemble mean SLR scenarios at each tide station based on the projections of Kopp et al., (2014)^23^. Each solid line corresponds to the SLR projection for a single tide station, and each station is colored according to its classification shown in Figure 1. The black solid lines in Extended Data Figure 2 correspond to a few tide stations (generally located at high latitudes) where SLR is projected to decrease with time in the next few decades due to local land uplift associated with sea-level fingerprinting. As in Extended Data Figures 1, the second column of Extended Data Figure 2 (e.g., panels B, F, and J) applies the empirical exceedance probability distribution resulting from all values of the recorded hourly water level. The third column of Extended Data Figure 2 (e.g., panels C, G, and K) applies the empirical exceedance distribution of the extremes, and the fourth column of Extended Data Figure 2 (e.g., panels D, H, and L) uses the best-fit GEV model for the exceedance distribution of the extremes. Once more, the x- and y-axes of the current figure are on linear and (base-two) logarithmic scales, respectively. Hence, relationships that follow a straight line correspond to exponential growth with time on the x-axis. The red, black, and green dashed lines correspond to a doubling of exceedance probability with every 1 yr, 5 yrs, and 25 yrs, respectively, according to Eq. (3). As in Extended Data Figures 1, the results shown in Extended Data Figure 3 columns three and four are bounded by a factor of 150, in contrast to the results for the relative odds increase shown below in Extended Data Figure 4.


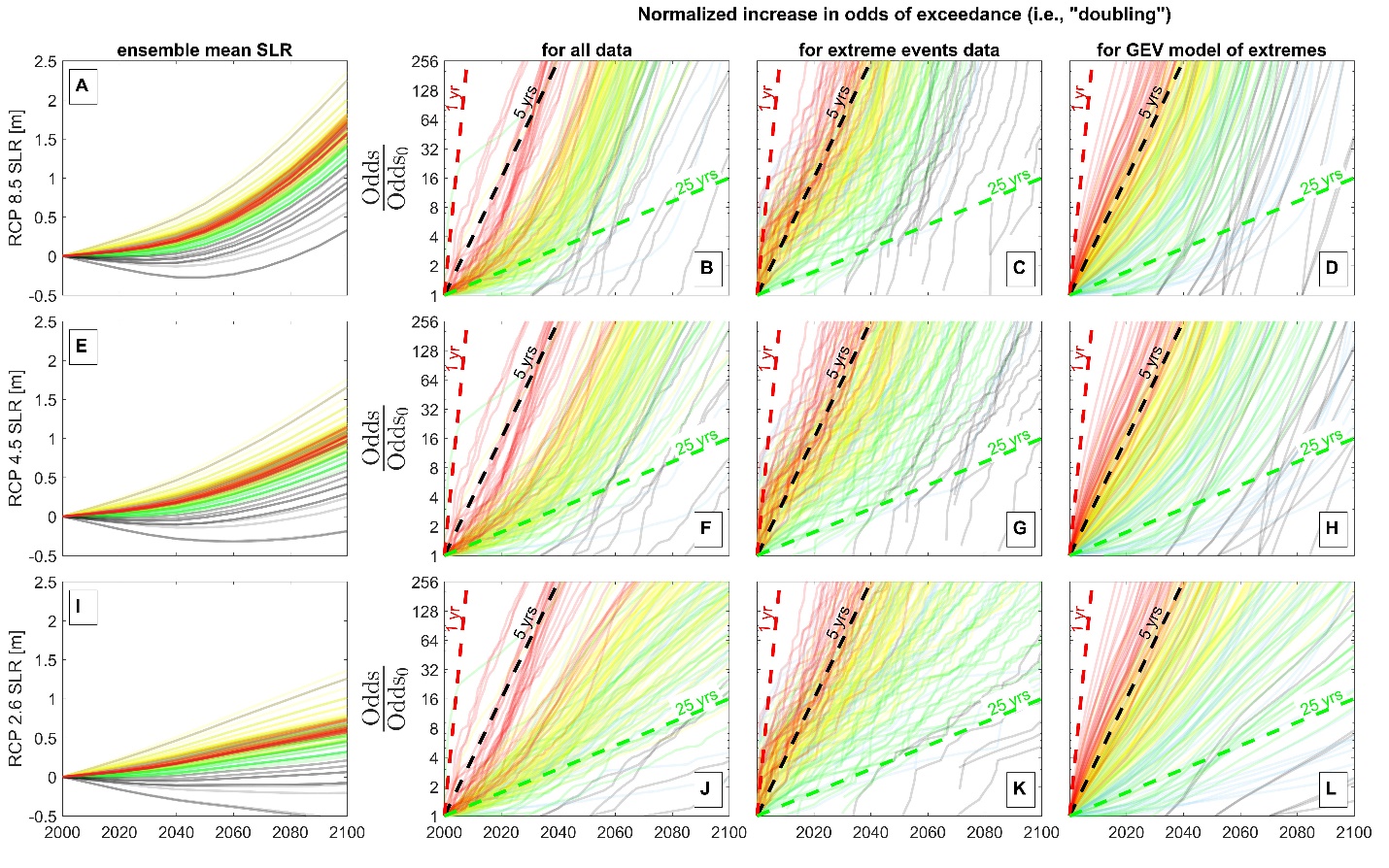


Extended Data Figure 4 - The relative increase in the odds of exceeding the present-day 50-year water-level event, $O/O_{0}$, with respect to time. As in Extended Data Figure 3, panels B, F, and J apply the empirical exceedance probability distribution resulting from all values of the recorded hourly water level. Panels C, G, and K apply the empirical exceedance distribution of the extremes, and panels D, H, and L use the best-fit GEV model for the exceedance distribution of the extremes. The x- and y-axes of the current figure are on linear and (base-two) logarithmic scales, respectively. Hence, relationships that follow a straight line correspond to exponential growth with time on the x-axis. Each solid line corresponds to the relationship between $O/O_{0}$ and SLR for a single tide station, and each station is colored according to its classification shown in Figure 1. The black solid lines correspond to tide stations where SLR is projected to decrease with time in the next few decades (which are located at high latitudes due to GIA). The red, black, and green dashed lines correspond to a doubling of exceedance probability with every 1 yr, 5 yrs, and 25 yrs, respectively, according to Eq. (3).

Extended Data Figure 4 illustrates the same relationships as in Extended Data Figure 3 but for the relative increase in the odds of exceeding the present-day 50-year water-level event, $O/O_{0}$ , with respect to time. Note that Figure 6 in the main body of the manuscript shows only columns one and two of Extended Data Figure 4.

The salient differences between Extended Data Figure 1 and Extended Data Figure 2 owing to the different method to calculate the exceedance distribution are reflected in Extended Data Figure 3 and Extended Data Figure 4. Most obviously, growth in the relative exceedance probability is bounded whereas the growth in the relative odds of exceedance is not. Secondly, the curves derived from the empirical distributions (columns 2 and 3) are jagged compared to the smooth GEV distribution (column 4). Thirdly, the curves of the most vulnerable sites (in red) derived from the extreme value distributions (columns 3 and 4) appear slightly steeper than the ‘all-values’ distribution (column 2). This is again perhaps due to the limited variance among the extremes data. On the other hand, the least vulnerable sites (in blue and green) seem slightly less steep and more spread out for the extreme value distributions (columns 3 and 4) compared to than the ‘all-values’ distribution (column 2). Despite the differences between Extended Data Figures 1- 4, the rates of growth are remarkably similar among the different methods to calculate the exceedance probability as would be expected for a robust result. Further, the rates of growth are remarkably consistent across the different SLR projections (e.g., RCP 2.6, 4.5, and 8.5) for the most vulnerable sites (shown in red). On the other hand, the lower-emission SLR scenarios do seem to result in much slower growth rates for the lower vulnerability sites (in blue and green).


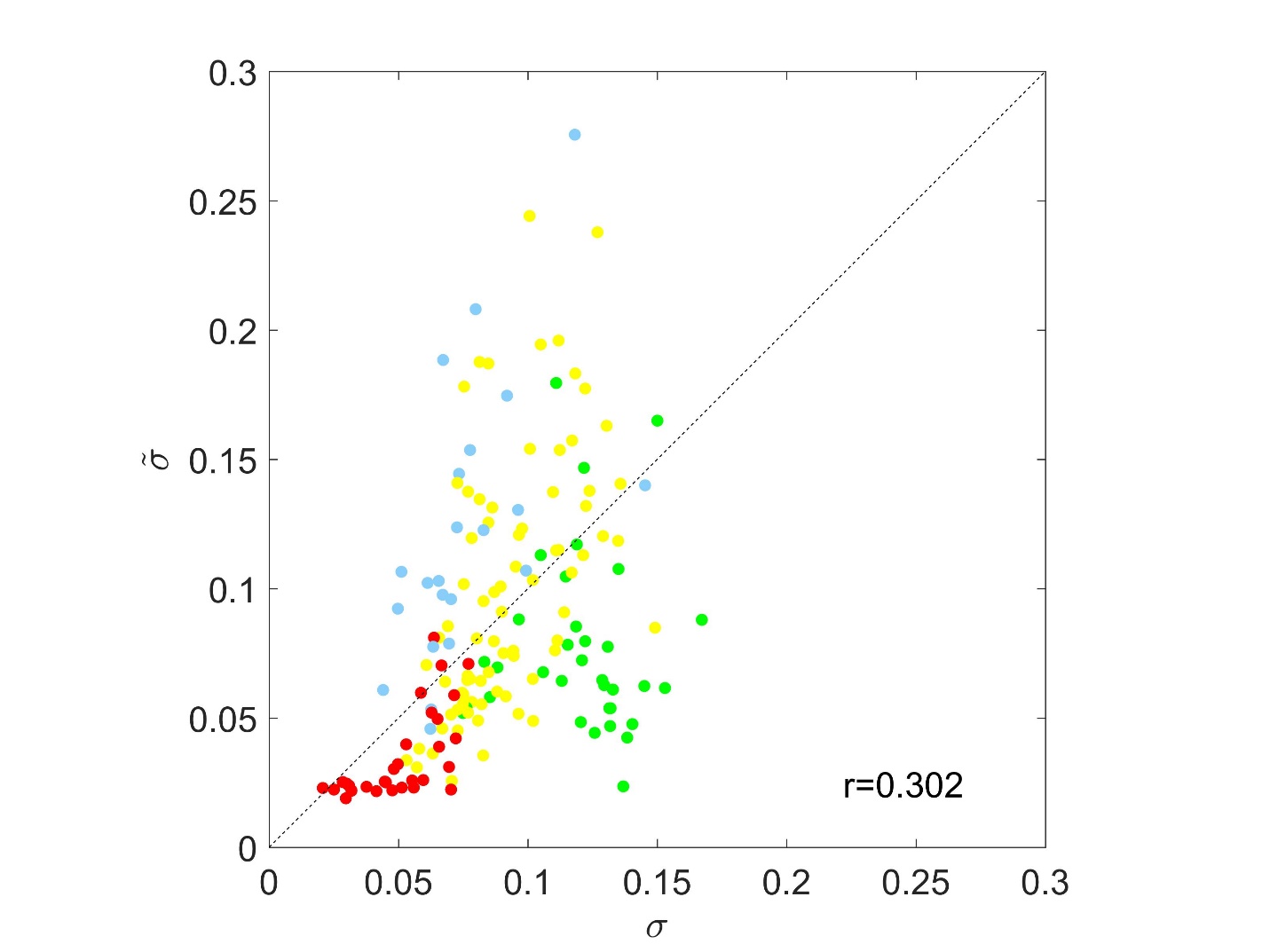


Extended Data Figure 5 – The relationship between the GEV scale parameter, $\sigma$ , and the amount of SLR that doubles the odds of exceeding the present-day 50-year water-level event, $\tilde{\sigma}$ , according to Eq. (5), at the tide stations used in the current study. Each station is colored according to its classification shown in Figure 1. Note that for the more vulnerable stations shown in red and yellow, the relationship between $\sigma$ and $\tilde{\sigma}$ is stronger than for the stations shown in blue and green. Overall, the correlation between $\sigma$ and $\tilde{\sigma}$, $r=0.302$ , is modest.

Applying the Gumbel distribution ($k=0$), Hunter (2012)^15^ predicted the growth in the expected number of exceedance events to be $\exp\left(\mu_{\text{SL}}/\sigma\right)$ and thus the growth rate is $\sigma$, the scale parameter of the Gumbel distribution. Here, we consider an alternate form of growth $2^{\mu_{\text{SL}}/\tilde{\sigma}}$ (based on Eqs. 3 and 5 in Methods) that seeks to be independent of any particular statistic model and seeks to represent exponential growth in a simple manner (i.e., via doubling). Hence, both $\sigma$ and $\tilde{\sigma}$can represent rates of growth, however, as shown in Extended Data Figure 5, they are not the same, although they bear some resemblance to each other. As has been explored in previous works (Buchanan et al., 2017)^16^, the rates of growth of the form considered Hunter (2012)^15^ are highly dependent on the family type of the GEV distribution (value of $k$), yet Hunter (2012)^15^ only considered the form of growth assuming $k=0$. As shown in Extended Data Figure 5, the growth rate is indeed highly dependent on the nature of extreme events reflected in the parameters of the GEV distribution, particularly the shape parameter $k$ (note the effect of the stations colored in blue). Yet, we find that the fairly simple doubling relationship ($2^{\mu_{\text{SL}}/\tilde{\sigma}}$) can accurately describe the rates of growth in exceedance due to SLR and the effects spanning the range of parameter space in the behavior of extremes (see e.g., Figure 4 and its corresponding discussion).
